# Nasopharyngeal Dysbiosis Precedes the Development of Lower Respiratory Tract Infections in Young Infants, a Longitudinal Infant Cohort Study

**DOI:** 10.12688/gatesopenres.13561.1

**Published:** 2022-04-12

**Authors:** Rotem Lapidot, Tyler Faits, Arshad Ismail, Mushal Allam, Zamantungwak Khumalo, William MacLeod, Geoffrey Kwenda, Zachariah Mupila, Ruth Nakazwe, Daniel Segrè, William Evan Johnson, Donald M Thea, Lawrence Mwananyanda, Christopher J Gill

**Affiliations:** 1 Pediatric Infectious Diseases, Boston Medical Center, Boston, MA, 02118, USA; 2 Pediatrics, Boston University School of Medicine, Boston, MA, 02118, USA; 3 Computational Biomedicine, Boston University School of Medicine, Boston, MA, 02118, USA; 4 Sequencing Core Facility, National Institute for Communicable Diseases, Johannesburg, 2131, South Africa; 5 Department of Veterinary Tropical Diseases, University of Pretoria, Pretoria, 0002, South Africa; 6 Department of Global Health, Boston University School of Public Health, Boston, MA, 02118, USA; 7 Department of Biomedical Sciences, School of Health Sciences, University of Zambia, Lusaka, Zambia; 8 Right To Care, Lusaka, Zambia; 9 Department of Pathology and Microbiology, University Teaching Hospital, Lusaka, Zambia; 10 Bioinformatics Program and Biological Design Center, Boston University, Boston, MA, 02118, USA; 11 Department of Biology, Boston University, Boston, MA, 02118, USA; 12 Department of Biomedical Engineering, Boston University, Boston, MA, 02118, USA; 13 Department of Physics, Boston University, Boston, MA, 02118, USA

**Keywords:** Lower Respiratory Tract Infection, Nasopharyngeal Microbiome, Dysbiosis, Longitudinal Cohort study

## Abstract

**Background:** Infants suffering from lower respiratory tract infections (LRTIs) have distinct nasopharyngeal (NP) microbiome profiles that correlate with severity of disease. Whether these profiles precede the infection or a consequence of it, is unknown. In order to answer this question, longitudinal studies are needed.

**Methods:** We conducted an analysis of a longitudinal prospective cohort study of 1,981 Zambian mother-infant pairs who underwent NP sampling from one-week through 14-weeks of age at 2-3-week intervals. Ten of the infants in the cohort who developed LRTI were matched 1:3 with healthy comparators. We completed 16S rRNA gene sequencing on the samples each of these infants contributed, as well as from baseline samples of the infants’ mothers, and characterized the normal maturation of the healthy infant NP microbiome, compared to infants who developed LRTI.

**Results:** The infant NP microbiome maturation was characterized by transitioning from *Staphylococcus* dominant to respiratory-genera dominant profiles during the first three months of life, similar to what is described in the literature. Interestingly, infants who developed LRTI had NP dysbiosis before infection, in most cases as early as the first week of life. Dysbiosis was characterized by the presence of *Novosphingobium, Delftia*, high relative abundance of *Anaerobacillus, Bacillus*, and low relative abundance of *Dolosigranulum,* compared to the healthy controls. Mothers of infants with LRTI also had low relative abundance of *Dolosigranulum* in their baseline samples compared to mothers of infants that did not develop an LRTI.

**Conclusions:** Our results suggest that NP microbiome dysbiosis precedes LRTI in young infants and may be present in their mothers as well. Early dysbiosis may play a role in the causal pathway leading to LRTI or could be a marker of other pathogenic forces that directly lead to LRTI.

## Background

Lower respiratory tract infections (LRTI), including pneumonia and bronchiolitis, are the leading cause of death in children under five years of age, accounting for 1.3 million deaths each year, with 81% concentrated in children 2 years or younger ( Cao *et al.*, 2019; Fischer Walker *et al.*, 2013). A necessary step leading to LRTI is the acquisition of a respiratory pathogen, such as *Streptococcus pneumoniae*. However, pneumococcal carriage is nearly universal among infants, only a few of whom develop severe invasive disease ( Balsells *et al.*, 2018; Yildirim *et al.*, 2010; Yildirim *et al.*, 2017). This indicates that the presence of the pathogen, while necessary, does not adequately address the more fundamental question of why some infants develop LRTI while most do not. 

Increasingly, LRTI is seen as a consequence of the interaction between the pathogen and other contextual factors. Such factors include the net immune state of the host, intercurrent viral infections that may act transiently, or, in the case of HIV, for extended periods. Another factor may also be the microbial ecosystem in which the pathogen exists, *i.e.*, the nasopharyngeal microbiome. Looking at the microbiome as an ecosystem model considers individual members of that ecosystem to exist in some dynamic equilibrium characterized by reciprocal loops of interaction. As such, the interaction between the microbiome and a specific potential pathogen (i.e., a pathobiont), could influence the behavior of that pathogen to either impede or promote LRTI ( Brugger *et al.*, 2016; Stewart *et al.*, 2017).

In support of this ecosystem model, several cross-sectional studies have found that children with LRTIs often have distinct nasopharyngeal (NP) microbiome profiles at time of infection compared with healthy children. The NP microbiome profiles appear to be dominated by bacterial genera that differ between respiratory infections and health. For example, NP microbiomes dominated by *Streptococcus* and *Haemophilus* are associated with LRTI, whereas microbiomes profiles dominated by *Moraxella*, *Corynebacterium* and/or *Dolosigranulum* characterize healthy children. Further, NP microbiome characteristics correlate with the severity of respiratory disease and with clinical outcomes ( de Steenhuijsen Piters *et al.*, 2015; Hasegawa *et al.*, 2017). While provocative, such observations largely rest on cross-sectional studies, and so cannot resolve the direction of cause and effect: we do not know whether these microbial profiles are a result of the infection or whether they preceded it. If the latter is true, then differences in the NP microbiome could potentially represent a state of vulnerability, participating in a causal pathway leading to LRTI. 

To draw such inferences, it is necessary to have longitudinal data, with sampling of infants before the development of the LRTI. Since LRTI is a rare event, collecting longitudinal data is complicated by the large number of infants needed to be followed. Between 2015 and 2016, our team conducted a prospective cohort study and was able to create a biological sample library that allowed a longitudinal analysis of this kind. The study took place in Lusaka, Zambia, among 1,981 mother-infant pairs: The Southern Africa Mother Infant Pertussis Study – SAMIPS ( Gill *et al.*, 2016). The pairs were enrolled one-week post-partum. All enrolled infants were healthy and born term. At baseline, and every two-three weeks thereafter through 14 weeks of age, we obtained NP samples from mother and baby. 

Within this cohort of 1,981 healthy infants a sub-set of 10 infants developed LRTI based on standard WHO clinical criteria ( *Revised WHO classification and treatment of childhood pneumonia at health facilities • EVIDENCE SUMMARIES •*, 2014), and adjusted for the purpose of this analysis. By comparing the infants who developed LRTI to matched healthy infants, we were able to conduct a time series analyses of NP microbiome of both infant populations, using 16S ribosomal DNA sequencing. We focused on the following fundamental analyses: 1) what is the ‘normal’ pattern of NP microbiome maturation over the first several months of life? 2) how does this contrast with the maturation of NP microbiome of infants who developed LRTI? 3) is there evidence that NP dysbiosis precedes the onset of LRTI? 4) are there distinct microbiome profiles that characterize sickness and health and other infant characteristics? 5) Is there also evidence of NP dysbiosis among the mothers of infants who later developed LRTI?

## Methods

### Study population

This is a nested time-series case comparator study within the prospective longitudinal Southern Africa Mother-Infant Pertussis study (SAMIPS). SAMIPS was a study conducted in Zambia in which infants and their mothers were followed over the first three months of life. Full methods description is previously detailed by Gill *et al.* ( Gill *et al.*, 2016), in short: All infants enrolled to SAMIPS were less than ten days of age, born term, via normal vaginal delivery, and deemed healthy after birth. All infants received scheduled vaccines. Immunization schedule in Zambia includes the Baccilus Calmette-Guèrin (BCG) vaccine at birth. The Diphtheria, Tetanus and whole cell pertussis vaccine, H. influenza B, and Hepatitis B vaccines (DTwP-Hib-HepB), the 10-valent pneumococcal conjugate vaccine (PCV10) and the Oral Poliomyelitis Vaccine (OPV) at one month, two months and three months of age. The inactivated polio vaccines is scheduled at 3 months of age, and the 2 doses of Rotavirus vaccine at one and two months of age. Written informed consent was obtained as appropriate from mothers of infants enrolled in the study.

The study was approved by the ethical review committees at the ERES Converge IRB in Lusaka, Zambia, and at Boston University Medical Center. All mothers provided written informed consent, with consent provided in English, Bemba or Nyanja as preferred by the participant.

### Study design

Mother-infant pairs were enrolled when mothers returned for their first postpartum well-child visit at one week of age. At enrollment, and 2–3-week intervals thereafter, through 14 weeks, we obtained a posterior nasopharyngeal (NP) swab from both mother and baby, with additional swabs obtained adventitiously if either returned seeking care for an acute respiratory infection. 

Within the SAMIPS cohort, we identified ten infants who during the study period suffered from symptoms of lower respiratory tract infection (LRTI) as adopted from the WHO ( *Revised WHO classification and treatment of childhood pneumonia at health facilities • EVIDENCE SUMMARIES •*, 2014). Since the aim of the WHO classification is to identify all possible cases of LRTI/pneumonia and guide management and treatment of these infants, the sensitivity of the constellation of symptoms is high. To increase the specificity of the diagnosis of LRTI (and pneumonia) for our study purpose, we adjusted the WHO definition of LRTI to a more conservative definition, including infants who had both high respiratory rates and indrawing of the chest. In our cohort there were ten sick infants that met our adjusted criteria, that were matched 1:3 with healthy comparators by season of enrollment, maternal age and household composition. All longitudinal samples of infants in our cohort, as well as the sample taken from the mothers at the first study visit were included in the microbiome analysis.

### Sample processing and storage

NP swabs were obtained from the posterior nasopharynx using a sterile flocked tipped nylon swab (Copan Diagnostics, Merrieta, California). The swabs were then placed in universal transport media, put on ice and transferred to our onsite lab on the same campus, where they were aliquoted and stored at -80°C until DNA extraction. DNA was extracted using the NucliSENS EasyMagG System (bioMérieux, Marcy l’Etoile, France). Extracted DNA was stored at our lab located at the University Teaching Hospital in Lusaka at -80°C. Sample collection, processing and storage were previously described ( Gill *et al.*, 2016).

### 16S ribosomal DNA amplification and MiSeq sequencing.

For 16S library preparations, two PCR reactions were completed on the template DNA. Initially the DNA was amplified with primers specific to the V3–V4 region of the 16S rRNA gene ( Klindworth *et al.*, 2013). The 16S primer pairs incorporated the Illumina overhang adaptor (16S Forward primer

5’-TCGTCGGCAGCGTCAGATGTGTATAAGAGACAGCCTACGGGNGGCWGCAG-3’;

16S reverse primer

5’-GTCTCGTGGGCTCGGAGATGTGTATAAGAGACAGGACTACHVGGGTATCTAATCC-3’)

Each PCR reaction contained DNA template (~12 ng), 5µℓ forward primer (1μM), 5 µℓ reverse primer (1μM), 12.5 µℓ 2 X Kapa HiFi Hotstart ready mix (KAPA Biosystems Woburn, MA), and PCR grade water to a final volume of 25µℓ. PCR amplification was carried out as follows: heated lid 110°C, 95°C for 3 min, 25 cycles of 95°C for 30s, 55°C for 30s, 72°C for 30s, then 72°C for 5 min and held at 4°C. Negative control reactions without any template DNA were carried out simultaneously.

PCR products were visualized using Agilent TapeStation (Agilent Technologies, Germany). Successful PCR products were cleaned using AMPure XP magnetic bead-based purification (Beckman Coulter, IN). The IDT for Illumina Nextera DNA UD Indexes kit (Illumina, San Diego, CA) with unique dual index adapters were used to allow for multiplexing. Each PCR reaction contained purified DNA (5 μℓ), 10 μℓ index primer mix, 25 μℓ 2X Kapa HiFi Hot Start Ready mix and 10 μℓ PCR grade water. PCR reactions were performed on a Bio-Rad C1000 Thermal Cycler (Bio-Rad, Hercules, CA) Cycling conditions consisted of one cycle of 95°C for 3 min, followed by eight cycles of 95°C for 30 s, 55°C for 30 s and 72°C for 30 s, followed by a final extension cycle of 72°C for 5 min. PCR products of negative controls were confirmed negative on Agilent TapeSataion (no band observed).

Prior to library pooling, the indexed libraries were purified with Ampure XP beads and quantified using the Qubit dsDNA HS Assay Kit (Thermo Fisher Scientific, Waltham, MA). Purified amplicons were run on the Agilent TapeStation (Agilent Technologies, Germany) for quality analysis before sequencing. The sample pool (2 nM) was denatured with 0.2N NaOH, then diluted to 4 pM and combined with 10% (v/v) denatured 20 pM PhiX, prepared following Illumina guidelines. Samples were sequenced on the MiSeq sequencing platform at the NICD Sequencing Core Facility, using a 2 x 300 cycle V3 kit, following standard Illumina sequencing protocols. Negative controls were sequenced as well, resulting in extremely low reads that were not analyzed further.

Sequencing data were processed using QIIME2 ( Bolyen *et al.*, 2019) and Pathoscope2 ( Hong *et al.*, 2014) . Samples with less than 10,000 reads were excluded from further analysis.

To account for reagent contamination, in addition to the negative controls, we processed all samples in random, blinding for timing of collection as well as clinical data.

### Data processing

We assessed the quality of the sequencing data using FastQC ( Andrews, 2010), which indicated that the overall sequencing quality was excellent, with mean Phred quality scores remaining greater than 30 (>99.9% accuracy) for over 200bp for both forward and reverse reads. We used *Trimmomatic* ( Bolger *et al.*, 2014) to trim Illumina adapters and remove low-quality sequences, setting the tool’s parameters to LEADING:6, TRAILING:6, SLIDINGWINDOW:6:15, and MINLEN:36. This quality filtering removed less than 0.5% of reads from each sample.

We used PathoScope 2 to assign sequencing reads to bacterial genomes. We used all of RefSeq’s representative bacterial genomes (downloaded November 2, 2018) as a PathoScope reference library. From PathoScope’s subspecies-level final best hit read numbers, we compiled counts tables and relative abundance tables for each sample at the phylum, genus, and where possible, to the species level.

### Data and statistical analysis

***NP microbiome characteristics and evolution over time*.** We describe the normal evolution of the NP microbiome in healthy infants over the first three months of life. We calculated microbial richness using Chao1 index, and diversity of microbial taxa using the Shannon diversity index. We report the individual evolution of NP microbiome of each of the 10 infants who develop LRTI. In order to establish statistical significance, we used the *lmer* function from the *lme4* package for R ( Bates *et al.*, 2015) to apply a mixed-effects linear model to the log counts per million (logCPM) value of each genus, including age and HIV exposure as fixed effects and the study subject as a random effect. All p-values generated by these linear models are reported after False Discovery Rate (FDR) adjustment for multiple comparisons using the Benjamini-Hochberg method ( Benjamini & Hochberg, 1995). We only generated mixed-effects models for genera which had an average relative abundance of at least 0.5% across all healthy infant samples.

For visualization of the development of healthy NP microbiota, we grouped all infant samples by age (in days) into 7 bins, each comprising a 16-day age window (0–15 days, 16–31 days, etc). We only visualized genera which had an average relative abundance of at least 1% across all samples. The relative abundances of all genera which did not meet this threshold were summed into a group labelled “Other/Low abundance” for plotting purposes only.

We calculated estimates of the alpha diversity within each sample based on the species-level counts tables generated by PathoScope 2. We calculated alpha diversity using two methods: the Chao1 index, which estimates the total number of species present within a sample, and the Shannon index, an entropy-based metric which incorporates both the number of species present and the evenness of abundance among those species. The Chao1 index was calculated using the R package *fossil* ( Vavrek, 2011) and the Shannon index was calculated using the R package *vegan* (available via CRAN) ( Oksanen *et al.*, 2019) each with a rarefaction depth of 10,000. We constructed a mixed-effects linear model as described above, except using each alpha diversity metric as a response variable, in order to test whether alpha diversity changed as infants aged.

***Analysis of the association between the NP microbiome and the development of LRTI*.** We used the lmer function from the *lme4* package (described above) to build mixed-effects linear models to compare the development of the NP microbiomes of infants who developed LRTIs to those of healthy infants. This time, we included infection status and the interaction of infection status with age as fixed-effect covariates in addition to age and HIV exposure, as well as study subject as a random effect. Once again, p-values were generated using the Anova function of the *car* package ( Fox & Weisberg, 2019) and then FDR corrected.

We similarly modified the models we had used to test alpha diversity in order to see if either Shannon or the Chao1 index values were different in LRTI infants, once again adding infection status and the interaction between infection status and age as fixed effects.

***Differential abundance analysis at first timepoints*.** We performed differential abundance between the first samples from healthy and LRTI infants using the R package *DESeq2* ( Love *et al.*, 2014) available via Bioconductor ( Huber *et al.*, 2015). We imported our unnormalized genus counts table compiled from PathoScope2 as a DESeqDataSet and ran the function DESeq, using a design model that included infants’ HIV exposure (from an HIV infected mother) as a covariate. For microbiome data, DESeq2 has been shown to return lower false discovery rates than other differential tests ( Mcmurdie & Holmes, 2014), and performs particularly well for smaller experiments ( Weiss *et al.*, 2017).

To test whether the presence or absence of certain genera at the first sampled timepoint were associated with LRTI, we performed Fisher’s exact test to determine if healthy and LRTI infants are equally likely to have each genus in their NP microbiome. Because very low-abundance genera could be the result of spurious alignments or contamination, we explored both a high threshold (>1% relative abundance) and a low threshold (>0.1% relative abundance) for defining presence of a genus.

***Beta diversity and clustering*.** We computed a Bray-Curtis dissimilarity matrix between samples using *vegan*’s vegdist function. When applied to relative abundance values, Bray-Curtis dissimilarity between two samples *i* and *j* is defined as $BC_{ij}=1-\sum_{n=0}^{N}\min(g_{in},g_{in})$ where *g _in_* is the relative abundance of genus *n* in sample *i*. We performed hierarchical clustering of samples based on this dissimilarity matrix using R’s hclust function with the method set to “ward.D”. We defined clusters using R’s cuttree function, with the value for k selected by maximizing the Silhouette and Frey indexes as calculated by the package *NbClust* ( Charrad *et al.*, 2014). For each cluster, we performed Fisher’s exact tests to determine whether that cluster was enriched for LRTI samples generally, pre-symptomatic samples, active symptom samples, or HIV-exposed samples.

We used the metaMDS function from the R package *vegan* to perform non-metric multidimensional scaling (NMDS) ordination on our Bray-Cutris dissimilarity matrix, using as parameters k=3, try=50, and trymax=1000. Scaling our data onto just two dimensions using NMDS yielded a stress value greater than 0.2, indicating a poor fit; we instead scaled the data onto three dimensions (stress=0.13), and used the vegan’s envfit function to project the age and LRTI status of each sample into the NMDS ordination. 2-dimensional plots of our NMDS ordinated data can be found in Figure 2, and a 3-dimensional plot can be found in Extended data Figure 4.

***Differential analysis of maternal NP microbiomes*.** We used Spearman correlation coefficients to verify that the composition of infant NP microbiomes is related to their mother’s NP microbiome. We chose Spearman correlation, which utilizes rank order rather than continuous values, due to the compositional nature of bacterial abundance data. We calculated Spearman’s ρ for the relative abundance of each genus between mothers and their infants. We tested the significant of these correlations by comparing the distribution of ρ values to 1000 null distributions of the same metric, generated by randomly permuting the mother/infant labels.

We used DESeq2 to test for differential abundance of genera in the NP microbiomes of mothers of LRTI infants and mothers of control infants. For this analysis, we only included samples taken from mothers at the earliest pediatric visits, before their infants began exhibiting LRTI symptoms. We included the HIV status of the mothers as a covariate in DESeq2’s regression model. We report p-values after FDR correction via Benjamini-Hochberg procedure, and consider adjusted p-values below 0.1 to be significant.

## Results

Within the SAMIPS cohort we identified 247 infants who developed LRTI during the study period as defined by the WHO clinical criteria: cough/cold symptoms AND fast breathing or chest indrawing ( *Revised WHO classification and treatment of childhood pneumonia at health facilities • EVIDENCE SUMMARIES •*, 2014). To increase the specificity of LRTI cases for our analysis we identified 10 infants that had both chest indrawing and fast breathing, most of which also had other danger signs (lethargy, poor feeding, vomiting or convulsions), and thus presented with severe LRTI. We then matched these case infants by season of birth, number of siblings and HIV exposure status, with healthy comparators. With ten infants with LRTI and 3:1 matching, our analysis set consisted of 40 infants at ~seven time points each. All infants were born healthy via vaginal delivery. Male sex was more common in infants who developed LRTI (p= 0.067). A third of infants with LRTI were born to mothers with HIV (receiving anti-retroviral treatment), compared to 40% of infants in the healthy group. Basic characteristics of the 40 infants are shown in Table 1. The symptoms and timing of sampling of the ten infants who developed LRTI are shown in Table 2.

**Table 1.** Characteristics of healthy infants and infants with LRTI.

Characteristics	Healthy Infants (N=30)	Infants with LRTI (N=10)	All Subjects (N=40)
Sex, n (%)			
Females	16 (53.3%)	2 (20.0%)	18 (45.0%)
Males	14 (46.7%)	8 (80%)	22 (55%)
Season of enrollment			
Dry Season (May-Oct), n (%)	28 (93.3%)	8 (80.0%)	36 (90.0%)
Rainy Season (Nov-Apr), n (%)	2 (6.7%)	2 (20.0%)	4 (10.0%)
Median age at enrollment in days (IQR)	7.0 (6 - 9)	7.0 (6 - 10)	7.0 (6 - 9)
HIV exposed, n (%)	13 (43.3%)	3 (30.0%)	16 (40.0%)
Mean number of samples collected (SE)	6.6 (0.2)	6.6 (0.6)	6.7 (0.2)

**Table 2.** Clinical symptoms and age of 10 infants with LRTI at each study visit/NP sampling.

Infants with LRTI	Sample Number (and age at sampling)								
	1	2	3	4	5	6	7	8	9
1.	7 days	27 days	42 days	62 days	79 days				
2.	7 days	27 days	35 days	42 days	59 days	73 days	88 days	104 days	
3.	7 days	11 days	62 days						
4.	7 days	19 days	45 days	60 days	68 days	107 days			
5.	7 days	28 days	42 days	56 days	69 days	84 days	100 days		
6.	7 days	21 days	42 days	56 days	59 days	73 days	87 days	96 days	103 days
7.	7 days	50 days	59 days	73 days	87 days	106 days			
8.	7 days	24 days	27days	42 days	61 days	73 days	90 days	104 days	
9.	7 days	24 days	39 days	44 days	65 days				
10.	7 days	23 days	40 days	61 days	83 days	99 days	113 days		

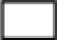
 No symptoms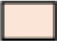 Mild upper respiratory symptoms (cough/runny or blocked nose)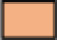 Diagnosis of LRTI (cough/runny or blocked nose with or without fever AN 0 fast breathing with indrawing of the chest)

### 16S ribosomal DNA amplicon sequencing data and processing

We successfully sequenced 265 NP swabs from 40 infants, capturing a median of seven samples from each infant. The median age at first sampling was seven days, and the median age at final sampling was 104 days. We also sequenced two NP swabs from each infant’s mother at first and last time points, for a total of 345 samples from mothers and infants combined. In six of these samples, fewer than 10,000 reads aligned to RefSeq reference genomes and were excluded from further analysis. The remaining 339 samples had a median of 101,979 reads per sample assigned to reference genomes and were included in the analysis. From these, we detected 421 unique genera, spanning 14 unique phyla, which were assigned at least 100 sequence reads across all samples. Based on these results, we were confident in our ability to proceed with the ensuing analyses.

### Analysis One: What is the NP microbiome maturation in healthy infants in the first three months of life?

Given our ultimate goal of identifying characteristics of the NP microbiome in infants who develop LRTI, as a first step we describe the characteristics and evolution of NP microbiome of the healthy infants. We analyzed the NP samples from all of the infants who remained free of LRTI through the end of observation, using linear regression to track changes in relative abundance of genera over time spanning the period between enrollment after birth and 14 weeks of age.

**Figure 1.**
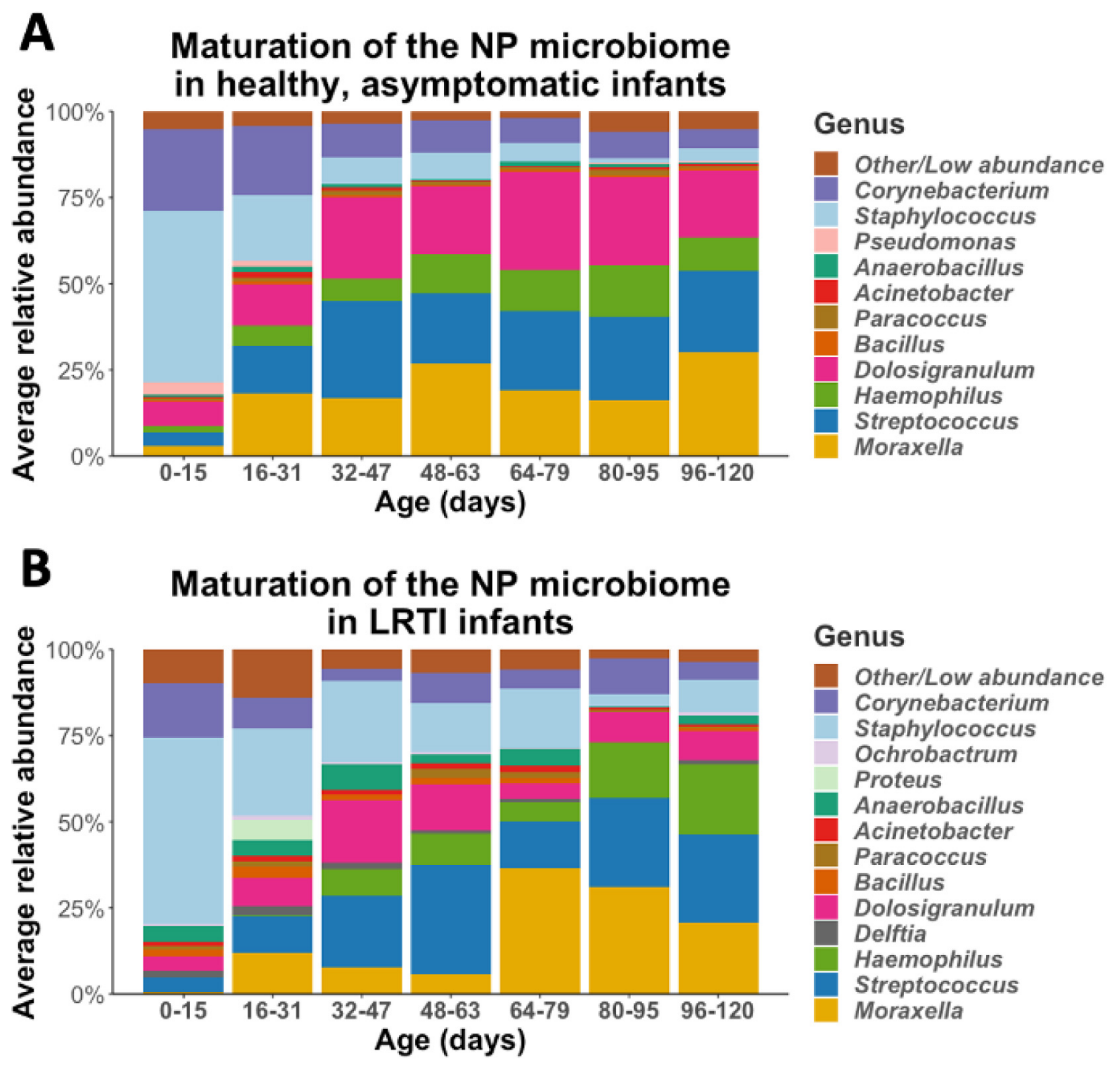
The maturation of the NP microbiomes of **A**) healthy, asymptomatic infants (n=30), and B) LRTI infants (n=10) over three months of observation. These stacked bar plots show the average relative abundance of the most common genera found in infant NPs, with samples binned by age.

We observed a stepwise pattern of maturation as the infants aged, summarized in Figure 1a, showing the relative abundance of different genera across each age averaged stratum. As can be seen, there is a clear shift in the abundance of dominant genera with time, with some dominating early in life, and others becoming more prominent as the children aged. Early in life, the dominant genera were *Staphylococcus* and *Corynebacteria*. According to a mixed-effects model, these genera declined in relative abundance as infants aged ( *Staphylococcus*: p<10E-7, *Corynebacteria*: p<0.001) and were replaced primarily by *Streptococcus* (p<0.001) *Dolosigranulum* (p<0.001), *Moraxella* (p<0.001), and *Haemophilus* (p=0.02). 

**Figure 2.**
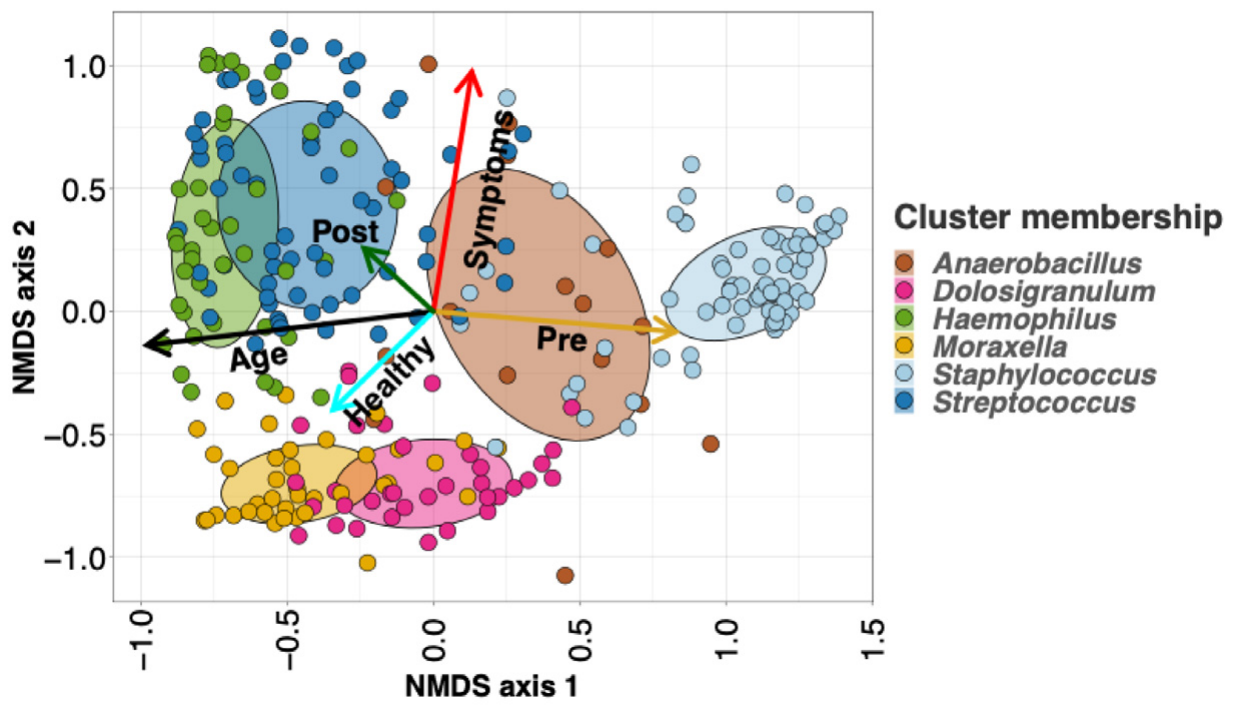
Nonmetric multidimensional scaling (NMDS) ordination plots of all infants’ (n=40) nasopharyngeal (NP) samples. We applied 3-dimensional NMDS ordination to the Bray-Curtis dissimilarity matrix between all infants’ NP swabs, and projected vectors into that ordination space representing the best fit correlations for the age at sampling (the black arrows) and LRTI status (the cyan arrows represent control infants). Age is highly correlated with the first NMDS axis, and samples on the young end of the age vector mostly belong to the *Staphylococcus*-dominated profile, whereas samples on the older end tend to belong more to the *Haemophilus* and *Moraxella*-dominated profiles. The *Dolosigranulum*-dominated profile is associated with the healthy end of the vector for LRTI status, while the *Anaerbacillus*-dominated profile is associated with disease

We did not measure any significant change in the alpha diversity (richness within a given sample) of NP microbiomes as healthy infants aged, measured either by Shannon index (p=0.32) or Chao1 index (p=0.15). However, alpha diversity only reflects the number of dominant genera, and not whether the dominant genera are themselves diverse. Thus, when we clustered samples based on beta diversity (between sample diversity), measured as the Bray-Curtis dissimilarity between pairs of samples, we identified a distinct profile associated with samples from very young infants that contrasted against several profiles for more mature infant NPs (Extended data Figure 1a). While each cluster is dominated by one or several of the most common genera, very few samples from healthy infants had high abundance of genera outside of the six most prominent genera. The primary axis of a Principal Coordinate Analysis (PCoA) (Extended data Figure 1b) correlated with the age of the infants at the time of sampling, and stratified samples mainly by relative abundance of *Staphylococcus* and *Corynebacterium* in younger infants vs. the genera which were more common at older ages. The second PCoA axis distinguished between samples that were rich in *Moraxella* or *Dolosigranulum* from those rich in *Streptococcus* or *Haemophilus*. In summary, this analysis showed that the microbiomes of early infancy were highly dynamic over time, but that these shifts occurred in a structured and stereotypical pattern.

### Analysis Two: Does the maturation of the NP microbiome differ among infants who developed LRTI compared with healthy infants?

Given evidence from prior literature that during LRTIs the NP microbiome of infants is different than that compared to healthy infants, we set out to test whether the general maturation pattern of the NP microbiome in the first months of life is altered in infants who went on to develop an LRTI. We repeated our analysis as described for healthy infants, stratifying into age groups and mapping the evolution of the NP microbiome over the first three months of life ( Figure 1b). Infants who developed LRTI had similar general succession patterns as described for healthy infants, with high relative abundance of *Staphylococcus* early in life replaced by relative abundance of *Streptococcus, Haemophilus, Corynebacterium, Dolosigranulum* and *Moraxella*. Even though the general succession pattern of NP microbiome in infants with LRTI were similar to succession patterns of healthy infants, they exhibited distinct characteristics. Notably, the NP microbiome of infants who developed LRTI had, on average, higher relative abundance of specific genera including *Bacillus* (p=0.05) and *Delfia* (p<0.001) and lower relative abundance of *Dolosigranulum* (p<0.001).

As with the healthy control infants in our analysis 1, we did not observe any change in alpha diversity in LRTI infants as they aged (Shannon: p=0.08, Chao1: p=0.74). Analysis of the beta diversity between LRTI infant samples once again revealed a cluster of samples taken at very early time points, dominated by *Staphylococcus*, with samples taken from older timepoints exhibiting profiles rich in *Streptococcus*, *Dolosigranulum*, *Moraxella*, and *Haemophilus.* However, in LRTI infants we also observed a large sixth cluster, characterized by a high abundance of *Anaerobacillus* as well as various other rare genera (Extended data Figure 2).

Since each infant developed an LRTI at a different age, stratifying the infants into age groups resulted in grouping together infants at different time points in relation to their disease – before the LRTI, at time of the LRTI, and following the LRTI. In order to describe maturation of the microbiome up to the time of LRTI, we created individual plots of the NP microbiome for each one of the ten sick infants (Extended data Figure 3). These emphasize the high degree of heterogeneity of patterns over time across individuals. 

### Analysis three: Does dysbiosis of the NP microbiome precede the development of LRTI?

A key limitation of the previous analyses is that they present the average across each time point, and since the LRTI event is occurring as one moves from left to right, more and more of the data in the LRTI group will represent the LRTI event or post LRTI timepoints. This is particularly important since all these children received antibiotics with their diagnosis, which will have obvious impacts on the microbiome. To address this source of confounding, we conducted several analyses. First, we present a comparison of the microbiomes for each group at the baseline visit, which preceded all LRTI events. Second, we present NMDS plots with individual vectors for pre-symptomatic, symptomatic, and post-symptomatic samples. Third, we present an indexed look back analysis. In this, we normalize our analysis by centering on the LRTI and examine the time points preceding the LRTI (for the case infants) and the same time points for the control infants. The limitation of this is that it removes the effect of age per se. The advantage is that age is controlled for by the matching. In this way, we can isolate the time periods preceding the LRTI as the points of greatest interest because they are most proximate to the incident.

We analyzed the earliest NP samples taken from each infant at 7 days of age, comparing the microbiomes of those infants who eventually developed LRTIs to those who did not. At enrollment all infants were healthy by definition (based on enrollment inclusion/exclusion criteria), and therefore, infants who developed LRTI could collectively be grouped as “prior to infection” at that time point.

We used the R package DESeq2 to perform differential abundance tests. To be considered significant, a given genus would need to be differentially abundant with an FDR-adjusted p-value of less than 0.1 and also a mean relative abundance of at least 0.1% among either healthy or sick infants. We identified three options by which a genus could be different between the 2 groups: First, a genus that was identified exclusively in infants who developed LRTI, such as *Novosphingobium* (4/10). Second, genera that were more common in infants with LRTI (but were present in both groups), such as *Delftia* (8/10 in LRTI infants vs 13/30 in healthy infants). And third, there were genera that were detected in both groups, but were present with higher relative abundance in infants with LRTI compared to the healthy infants, such as *Anaerobacillus*, *Bacillus*, *Blastococcus*, *Brachybacterium*, *Ochrobactrum*, *Ornithinimicrobium*, and *Sphingomonas*. Overall, ten genera were significantly different in infants who later developed LRTI at the first time point ( Table 3). Notably, *Dolosigranulum,* which has been identified in prior studies as being associated with a healthy microbiome *,* as was the case among the healthy infants here, had significantly lower relative abundance in infants who developed LRTIs than in healthy counterparts prior to the LRTI and even at the first sample time point.

**Table 3.** Differential abundance between control and LRTI infants at earliest observed timepoint.

Genus	Log Foldchange	Frequency in control infants	Frequency in LRTI infants	Adjusted p-value
*Anaerobacillus*	2.66	70%	70%	0.013
*Bacillus*	2.54	60%	70%	<0.01
*Blastococcus*	5.36	0%	10%	<0.01
*Brachybacterium*	5.22	3%	30%	<0.01
*Delftia*	2.81	43%	80%	<0.01
*Dolosigranulum*	-4.14	57%	50%	<0.01
*Novosphingobium*	6.80	0%	40%	<0.01
*Ochrobactrum*	2.62	27%	60%	<0.01
*Ornithinimicrobium*	4.77	3%	20%	<0.01
*Sphingomonas*	2.72	17%	40%	<0.01

Nonmetric multidimensional scaling (NMDS) scaling of the beta diversity dissimilarity matrix between all samples allows us to visualize more holistic structural differences in the NP communities of healthy vs LRTI infants ( Figure 2). When we project the age and eventual LRTI status of the infants into the NMDS ordination space, we can see that age correlates closely the primary NMDS axis, whereas LRTI status is mostly correlated with the secondary, indicating differences in NP microbiomes between healthy and LRTI infants independent of the aging process.

**Figure 3.**
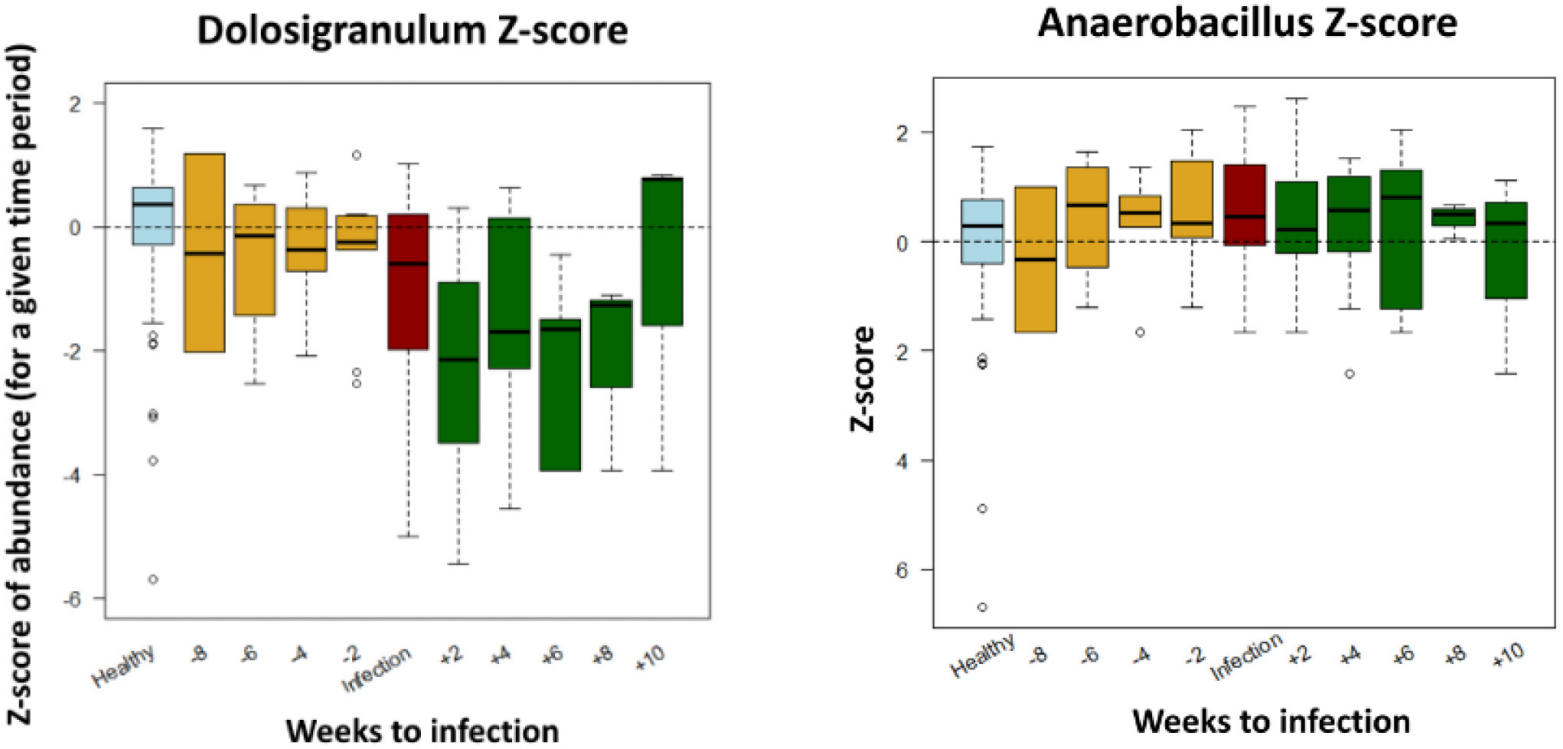
Relative abundance Z-scores of specific genera of LRTI infants compared to healthy controls (light blue), by weeks from infection.

Lastly, we looked into specific genera, comparing their abundance relative to the time of infection ( Figure 3). Concentrating on the samples taken prior to infection (and prior to antibiotic administration), this analysis confirmed lower relative abundance of *Dolosigranulum,* and higher relative abundance of *Anaerobacillus* in the LRTI infants before their infection.

### Analysis four: Are there distinct microbiome profiles that characterize sickness and health and other infant characteristics?

In order to identify specific microbial profiles, we applied hierarchical clustering to the Bray-Curtis dissimilarity matrix between each pair of samples from all infants. The Bray-Curtis dissimilarity matrix is a common tool in ecology for measuring the distance between different populations in terms of beta-diversity, and is bounded between 0 and 1, spanning ‘no dissimilarity’ to ‘complete dissimilarity’. We calculated at the genus level, using the *hclust* package in R. We used the Silhouette and Frey clustering indexes (NbClust) to determine the optimal heights at which to trim the dendrogram produced by the hclust function in R, splitting our samples into six primary clusters (Silhouette index) and 13 sub-clusters (Frey index). These six primary profiles were then named after the dominant genus within each cluster (the highest relative abundance genus). This yielded the following clusters: *Staphylococcus* dominant *Streptococcus* dominant, *Moraxella* dominant, *Dolosigranulum* dominant, *Haemophilus* dominant, and *Anaerobacillus* dominant profiles, corresponding to six of the seven most abundant genera across all our samples, as shown in Table 4. *Corynebacterium* is the only highly-abundant genus that does not compose the majority (or plurality) of relative abundance within any cluster; instead of being dominant in a subset of samples, *Corynebacterium* often co-occurred alongside the more dominant *Staphylococcus*, or to a lesser extent *Dolosigranulum*. For ease of reporting, we shall henceforth refer to each cluster by its most abundant genus.

**Table 4.** Relative abundance and frequency of the most common genera observed in the NP microbiome of healthy control and LRTI infants.

Healthy infants			
Genus	Mean Relative Abundance	Frequency (Samples)	Frequency (Subjects)
Streptococcus	19.4%	93.4%	100%
Dolosigranulum	19.1%	84.3%	100%
Moraxella	18.3%	77.8%	100%
Staphylococcus	14.0%	88.9%	100%
Corynebacterium	12.0%	94.4%	100%
Paracoccus	0.95%	66.7%	100%
Acinetobacter	0.84%	66.2%	100%
Bacillus	0.55%	52.5%	100%
Anaerobacillus	1.1%	53.0%	96.7%
Pseudomonas	0.89%	38.9%	93.3%
Delftia	0.32%	37.9%	90%
Aeromonas	0.13%	26.3%	80%
Haemophilus	8.7%	38.4%	73.3%
Kocuria	0.11%	18.7%	63.3%
Ochrobactrum	0.16%	16.2%	53.3%
Escherichia	0.25%	8.1%	40%
Enterobacter	0.18%	9.6%	40%
Klebsiella	0.12%	6.6%	30%
LRTI infants			
Genus	Mean Relative Abundance	Frequency (Samples)	Frequency (Subjects)
Staphylococcus	22.1%	90.8%	100%
Streptococcus	18.8%	93.8%	100%
Moraxella	14.8%	80%	100%
Dolosigranulum	9.6%	58.5%	100%
Corynebacterium	8.3%	86.1%	100%
Anaerobacillus	3.9%	58.5%	100%
Bacillus	1.7%	64.6%	100%
Delftia	1.5%	63.1%	100%
Acinetobacter	1.4%	73.8%	100%
Pseudomonas	0.43%	46.2%	100%
Paracoccus	1.1%	52.3%	90%
Ochrobactrum	0.64%	43.1%	90%
Haemophilus	8.0%	38.5%	80%
Novosphingobium	0.37%	29.2%	80%
Kocuria	0.30%	26.2%	80%
Sphingomonas	0.38%	30.8%	70%
Aeromonas	0.17%	21.5%	60%
Janibacter	0.13%	12.3%	60%
Brachybacterium	0.11%	23.1%	60%
Agrobacterium	0.20%	15.4%	50%
Veillonella	0.19%	15.4%	50%
Cutibacterium	0.15%	12.3%	50%
Stenotrophomonas	0.14%	13.8%	50%
Halolactibacillus	0.11%	15.4%	50%
Proteus	0.92%	9.2%	40%
Nocardioides	0.12%	13.8%	40%
Variovorax	0.11%	12.3%	40%
Klebsiella	0.18%	7.7%	30%
Marmoricola	0.18%	9.2%	20%
Blastococcus	0.14%	6.2%	20%
Knoellia	0.11%	7.7%	20%
Anaerococcus	0.10%	6.2%	10%

**Figure 4.**
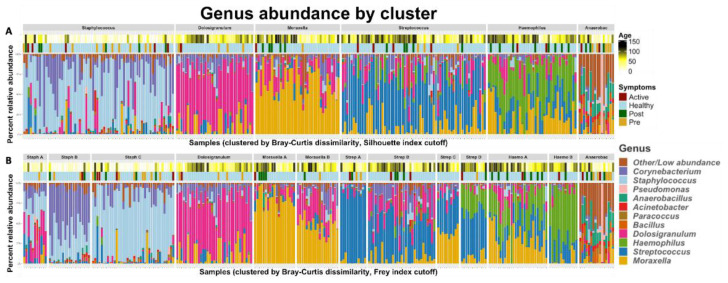
The taxonomic profiles of all infant NP samples (n=40), clustered by pairwise Bray-Curtis dissimilarity. Clusters were defined by performing hierarchical clustering on the beta diversity matrix and then cutting the resulting dendrogram into an optimal number of clusters according to the **A**) Silhouette index (6 clusters) and B) Frey index (13 clusters). The color bars above the stacked bar plots indicate the infants’ ages at the time of each sample and their LRTI status – “healthy” indicates an infant which did not develop LRTI symptoms during our observation.

Figure 4 shows the microbial composition of each of the 262 infant samples in our study which passed sample quality filters, grouped by the six primary profiles ( Figure 4A) and the 13 sub-profiles ( Figure 4B).

Fisher’s exact tests revealed that the *Anaerobacillus* dominant profile was highly associated with infants who developed LRTIs, (p<0.01, estimated odds-ratio=5.74). The *Staphylococcus* sub-profile Staph-C was associated with LRTI infants (p=0.04, estimated odds-ratio=2.26), and the *Streptococcus* subcluster Strep-C (which is also rich in *Moraxella*) was associated with healthy infants (p=.0.07). Using ANOVA to assess the association of each profile with age, the *Staphylococcus* dominant profile was clearly associated with samples from younger infants compared to all other profiles, and the *Anaerobacillus* dominant profile was associated with younger samples when compared to the *Haemophilus* and *Streptococcus* profiles ( Table 5).

**Table 5.** Associations between NP microbiome profiles with LRTI status and age.

Associations with LRTI status			
Cluster/Subcluster	Odds ratio estimate	Odds ratio range	P-value
Anaerobacillus	5.74	1.80-20.11	<0.01
Staphylococcus C	2.26	1.02-4.92	0.04
Streptococcus C	0.00	0.00-1.34	0.07
Associations with age (in days)			
Cluster	Cluster	Difference in age	Adjusted P-value
Staphylococcus	Moraxella	39	<0.01
Staphylococcus	Dolosigranulum	52	<0.01
Staphylococcus	Streptococcus	41	<0.01
Staphylococcus	Haemophilus	44	<0.01
Staphylococcus	Anaerobacillus	21	0.05
Anaerobacillus	Streptococcus	20	0.1
Anaerobacillus	Haemophilus	24	0.05

We visualized the association between LRTI status and NP taxonomic profiles using NMDS ordination ( Figure 2). By projecting infants’ LRTI status and age into the ordination space, we can see that the vector corresponding to healthy samples points towards the *Dolosigranulum* profile (and to a lesser extent towards the *Moraxella* profile), while the LRTI vector points towards the *Anaerobacillus* profile.

Together, these results reinforce a number of our previous observations; in particular, we can see that there is a general trend for infant NP microbiome profiles to shift from being *Staphylococcus* dominant shortly after birth towards several other profiles. We also see a clear dysbiotic pattern in the LRTI infants, comprising higher than normal relative abundance of *Anaerobacillus* as well as higher prevalence of rare genera which typically make up an extremely low portion of (or are completely absent from) healthy NP microbiomes.

### Analysis five: Is the NP microbiome of mothers of infants who develop LRTI different than mothers of healthy infants?

Observing distinct characteristics of the NP microbiome of infants as early as age 7 days, suggested that these profiles might be related to in-utero exposures, transmittable immunologic factors, and/or host genetics. That led us to question whether mothers of infants who develop LRTI have themselves distinct characteristics of the NP microbiome. We analyzed the first NP swabs from each of the mothers enrolled in our study taken at the infants’ day seven enrollment visits, correlated their microbiomes to those of their infants, and used DESeq2 to establish which genera were differentially abundant between mothers of LRTI infants and mothers of healthy infants. Similar to the pattern seen in the infants themselves, the mothers of infants who developed LRTIs had significantly decreased relative abundance of *Dolosigranulum* (p=0.05) as compared to mothers of healthy infants at 7 days of infant’s life. 

## Discussion

In this analysis, we show that the NP microbiome of infants with LRTI differs from that of healthy infants and that there is clear evidence of dysbiosis preceding the onset of LRTI. Intriguingly, we observed different microbiome patterns in the mothers of infants who later developed LRTI and those whose children remained healthy. That, and the fact that the microbiome of mother-infant pairs is more closely correlated within pairs than across pairs, suggests that some of the infant dysbiosis has transgenerational origins. As an overall synthesis, our data suggest that there are quantitative and qualitative differences between infants (and their mothers) who do and do not develop LRTI. This supports the hypothesis that LRTI is not a random event, but rather may reflect predispositions that are generally unobserved but may nonetheless play an essential or contributory role in the pathogenesis of childhood LRTI. 

The nasopharynx is the ecologic niche of respiratory pathobionts, and in this ecosystem they will either become invasive or remain merely colonizers. The NP microbiome at time of infection is associated with the risk of development of LRTI and its severity. But there is also good reason to believe that the maturation of the NP microbiome in the first months of life, and not only its characteristics at time of infection, is associated with respiratory health and development of disease later in life. For example, maturation of the gut microbiome is known to regulate the immune system evolution and is associated with the development of diseases later in life such as obesity and type 1 diabetes ( Bokulich *et al.*, 2016; Stewart *et al.*, 2018). Gut microbial dysbiosis in children often predisposes to recurrent *C. difficile* infections ( Ihekweazu & Versalovic, 2018). Thus, a similar association between the NP microbiome and risk of respiratory infections is a plausible theory for which there is precedent. 

We have shown that we can characterize the normal, healthy maturation of the NP microbiome over the first months of life, and how this maturation is different in infants who develop early LRTI. While the evolution of the normal microbiome is highly dynamic, it proceeds in a stereotypical fashion, with stepwise shifts from a flora dominated by skin organisms ( *Staphylococcus)*, to one that is dominated by genera more typically associated with the respiratory tract ( *Dolosigranulum, Streptococcus, Haemophilus and Moraxella).* Similar microbial succession patterns were previously described in other birth cohorts ( Biesbroek *et al.*, 2014).

By contrast, infants who develop LRTI have similar general succession patterns as healthy infants; transitioning form high relative abundance of *Staphylococcus* to high relative abundance of genera associated with the respiratory tract, but unlike healthy infants, the evolution of their NP microbiome is characterized by low relative abundance of specific genera associated with ‘health’, such as *Dolosigranulum*, and high relative abundance of other genera that appear unique, such as *Anaerobacillus, Bacillus,* and a mixture of ‘other’ uncommon genera. Additionally, the LRTI infants’ microbiomes include a larger number of uncommon and transient genera, presenting a picture that is more chaotic than what is seen in the healthy infants. Since the maturation analysis included post-LRTI samples, the differences observed in the NP microbiome maturation of LRTI infants could also be attributed to antibiotics and not only to the LRTI itself.

Case-control studies have consistently demonstrated an association between NP microbiome characteristics and LRTIs at time of disease, though interpretation in terms of causality could not be shown. The relatively high abundance of *Dolosigranulum/Corynebacterium* and *Moraxella* are correlated with healthy states ( Mansbach *et al.*, 2016), whereas NP microbiomes enriched with *Streptococcus* and *Haemophilus* are associated with LRTI and also correlate with severity of disease ( de Steenhuijsen Piters *et al.*, 2016; Kelly *et al.*, 2017). But are these microbial profiles a result of the infection? Or were they present before the infection?

We were able to identify several microbiome profiles which appear to cluster by chronological age, LRTI and health. Our results indicate that young infants who developed LRTI, had NP microbiome dysbiosis prior to acquiring the infection, and as early as 7 days of life. These infants have NP microbiome enriched with *Aneorobaccillus/Bacillus, Acinetobacter,* and other uncommon/unspecified genera, and also have relatively lower abundance of *Dolosigranulum*. Our intriguing results suggest that their mothers NP microbiome at the same early time point also differed from that of mothers of healthy infants.

The interaction between host, microbiome and pathobionts is complex and most probably multidirectional. The NP microbiome, known to be associated with environmental factors (breastfeeding, mode of delivery) ( Bosch *et al.*, 2017; Brugger *et al.*, 2016) could also very well be a reflection or marker of host genetics and immune system function, which would explain why so early in life “high risk” profiles are observed. New acquisition of a pathobiont in the nasopharynx initiates interactions between the pathobiont and other organisms residing in the nasopharynx. These interactions modify metabolic activity and gene expressions of the pathobiont that influence whether the pathobiont becomes invasive. The interactions themselves between organisms in the nasopharynx also modify host immune response which underscores the complex relationship between host, microbiome and pathogens ( de Steenhuijsen Piters *et al.*, 2019).

The key unresolved question is what role dysbiosis plays in the causal pathway leading to pneumonia: is dysbiosis a marker of other unobserved forces that lead to pneumonia, such as underlying host genetic or immunologic factors? Or does dysbiosis play a role in the causal pathway leading to LRTI? While our data cannot resolve this question, the implication of our findings are substantial. Our findings suggest that NP dysbiosis identified in the first days of life is associated with higher risk of developing LRTI in early infancy. This suggests that there is an important window of opportunity for identifying these infants and intervene. According to our findings, it may even be that we can identify these infants, by examining the mothers.

Our study has several limitations. Infants were followed until the age of three months, and thus our findings could not be generalized to older age groups. On the other hand, it is possible that infants included in our healthy control group developed LRTI after the study period, in that case our results are biased towards the null, possibly underestimating differences between the two groups.

A further limitation is that we do not know the causative pathogen of the LRTIs, and whether these were viral, bacterial, or mixed pathogen LRTIs. LRTI is a heterogeneous set of conditions, and it is plausible that dysbiosis can interact in pathogen-specific ways. The diagnosis of LRTI was based only on clinical data. Even though different pathogens interact in different ways with the NP microbiome and the host immune system, our data suggests that there is a common NP microbiome risk profile, regardless of the causative pathogen. Lastly, while our analysis included a very large number of longitudinal samples, our sample size only included 10 infants who developed LRTI (by our conservative definition). However, LRTI is a comparatively rare event and requires longitudinal surveillance of thousands of subjects over an extended period to identify even a few cases, which accounts for the paucity of research on this topic. Logistically, it is immensely challenging and resource intense to create and sample a cohort in the way we have done. Nonetheless, further research will be needed to confirm or refine these initial observations. If confirmed, these findings are not only critical to our understanding of factors that lead to the development of LRTI, and why one infant develops an LRTI while others do not, it also suggests that we have a window of opportunity to identify these “at-risk” infants before their infection, and to potentially intervene. These prevention measures could have a high impact on decreasing burden of LRTI in infancy.

## Conclusions

Dysbiosis of the NP microbiome in infants precedes LRTIs, suggesting at minimum a signal of infants at higher risk for LRTIs, and possibly a causative role in the development of these infections. Specific NP microbiome profiles which could be identified perinatally and appear to be associated with a higher risk of developing LRTIs in early infancy, present a potential window of opportunity for interventions. Our findings should be confirmed by large scale longitudinal studies.

## Declarations

**Ethics approval and consent to participate:** The study was approved by the ethical review committees at the ERES Converge IRB in Lusaka, Zambia, and at Boston University Medical Center. All mothers provided written informed consent, with consent provided in English, Bemba or Nyanja as preferred by the participant.

## Data availability

### Underlying data

GitHub. Infant_Nasopharyngeal_Dysbiosis. DOI: https://github.com/tfaits/Infant_Nasopharyngeal_Dysbiosis

This project contains the following underlying data:

- All code, processed data, and the sample information metadata
- Taxon counts tables are called "species.RDS", "genus.RDS", and "phylum.RDS". For strain/subspecies-level counts, "PathoScopeTable.txt" has the unfiltered/unprocessed outputs from PathoScope.

The raw and processed sequencing data from this study are available in the SRA repository, under NIH Sequence Read Archive, BioProject: PRJNA817266.

### Extended data

Harvard Dataverse. Nasopharyngeal Dysbiosis Precedes the Development of Lower Respiratory Tract Infections in Young Infants, a Longitudinal Infant Cohort Study. DOI: https://doi.org/10.7910/DVN/BWGTEQ ( Lapidot, 2022)

Data are available under the terms of the Creative Commons Zero "No rights reserved" data waiver (CC0 1.0 Public domain dedication).
