# Supplementary material for: Nasopharyngeal Dysbiosis Precedes the Development of Lower Respiratory Tract Infections in Young Infants, a Longitudinal Infant Cohort Study
Source: Gates Open Res. 2024 Mar 20;6:48. Originally published 2022 Apr 12. [Version 2] doi: 10.12688/gatesopenres.13561.2 (PMC11266592; doi:10.12688/gatesopenres.13561.2)
Supplement: Peer review history

# Reviewer report: Reviewer response for version 2

**DOI:** 10.21956/gatesopenres.16786.r36205

**Published:** 2024-07-23

**Authors:** Wouter A.A. De Steenhuijsen Piters

**Affiliations:** 1 Department of Paediatric Immunology and Infectious Diseases, Wilhelmina Children's Hospital, University Medical Center Utrecht, Utrecht, The Netherlands; 2 Centre for Infectious Disease Control, National Institute for Public Health and the Environment, Bilthoven, The Netherlands

We recognize the significance and novelty of the study, where the authors study the nasopharyngeal microbiota of Zambian infants, particularly those suffering from lower respiratory tract infections (LRTIs), in a longitudinal context. However, as was also discussed in our initial review, we still have two major concerns, which we believe are unsatisfactorily addressed.

 First and foremost, we fully agree with the concerns raised by the Reviewer 3 (Carter Merenstein) regarding potential contamination issues. The absence of laboratory controls (especially DNA isolation negative controls) raises doubts about the validity of the lab procedures and possibility of contamination. Additionally, the authors do not report on a measure for bacterial biomass, which could have provided crucial insights in the extent of contamination. This issue is of relevance since, as also Reviewer 3 notes, some of the genera described to be associated with LRTIs (including those making up the disease-associated *Anaerobacillus*-cluster) have not been described as part of the nasopharyngeal microbiome previously, instead being mentioned as contaminants in literature. This may also be the case for *Delftia*, *Bacillus*, *Paracoccus*, *Acinetobacter*, *Proteus* and *Ochrobactrum*.

 As said, we believe the study is of interest for the reasons stated in the introduction of our review. At the same time, we recognize this is a retrospective microbiome-study, where samples that were previously collected for a different purpose were sequenced at a later point in time, which brings many technical challenges, some of which cannot be amended. While the authors briefly acknowledge contamination as a limitation in their discussion (in a rather imprecise statement), we believe its importance has not been adequately emphasized throughout the article and - importantly - may not be clear to novice readers. At the very least, the likelihood of contamination should be underscored more prominently, particularly in the methods, results and discussion sections (see also major remark 1 in our initial review).

 Our second concern revolves around the title and analysis three. The title appears to derive from the results of analysis three, depicting the fluctuation in the relative abundance of *Dolosigranulum* and *Anaerobacillus* before (see notes on *Anaerobacillus* above), during and after LRTI infections. We would like to reiterate our previous remarks (major remark 2 and minor remark 42) regarding the lack of statistical support for these findings and the failure to account for the influence of age (especially when considering the *Dolosigranulum *dynamics). Given the absence of statistical validation and the inability to adjust for age in analysis three, we argue that the title does not appropriately capture the contents of the manuscript. Here, we feel that the authors did not sufficiently address our previous remarks and would urge them to reconsider addressing these concerns.

 Minor comments:

- Still unclear if PathoScope2 has been validated in the context of 16S-microbiota analyses
- Still unclear why the authors did not look at ASV/OTU level results
- Still use “evolution” instead of dynamics or development
- “Samples of all infants (both cases and comparators) were processed at the same time and under similar conditions, lowering the likelihood of contamination impacting the results” à processing the samples together does not necessary combat contamination; this merely standardizes the environmental factors during sample handling and processing.
- The genera in table 3 are all common contaminants
- It is unclear how the genera in Table 4 were selected? Top? I would suggest to include this in the table caption

Apart from these minor comments, there is also a significant number of (mostly minor) comments in our previous review the authors were not yet able to address. We would again urge them to critically go through our previous comments and see what can be done to take away our concerns/answer our questions.

Is the work clearly and accurately presented and does it cite the current literature?

Partly

If applicable, is the statistical analysis and its interpretation appropriate?

No

Are all the source data underlying the results available to ensure full reproducibility?

Yes

Is the study design appropriate and is the work technically sound?

Partly

Are the conclusions drawn adequately supported by the results?

No

Are sufficient details of methods and analysis provided to allow replication by others?

No

Reviewer Expertise:

Our group's expertise is on early-life development of respiratory microbiota in the context of health and disease. Both reviewers specialise in microbiota data analysis.

I confirm that I have read this submission and believe that I have an appropriate level of expertise to confirm that it is of an acceptable scientific standard, however I have significant reservations, as outlined above.

# Reviewer report: Reviewer response for version 2

**DOI:** 10.21956/gatesopenres.16786.r36206

**Published:** 2024-04-13

**Authors:** Frank A Scannapieco

**Affiliations:** 1 University at Buffalo, NY, USA

No further comments.

Is the work clearly and accurately presented and does it cite the current literature?

Partly

If applicable, is the statistical analysis and its interpretation appropriate?

I cannot comment. A qualified statistician is required.

Are all the source data underlying the results available to ensure full reproducibility?

Yes

Is the study design appropriate and is the work technically sound?

Partly

Are the conclusions drawn adequately supported by the results?

Partly

Are sufficient details of methods and analysis provided to allow replication by others?

Yes

Reviewer Expertise:

Oral microbiology; Relationships between oral disease and systemic disease.

I confirm that I have read this submission and believe that I have an appropriate level of expertise to confirm that it is of an acceptable scientific standard.

# Reviewer report: Reviewer response for version 2

**DOI:** 10.21956/gatesopenres.16786.r36204

**Published:** 2024-04-12

**Authors:** Carter Merenstein

**Affiliations:** 1 University of Pennsylvania, Pennsylvania, USA

This study leverages a prospective longitudinal cohort to identify changes in the nasopharyngeal microbiome that may precede the development of a lower respiratory tract infection (LRTI) in infants under 3 months. The prospective nature of this cohort is valuable, and provided the unique opportunity to separate causes and effects of LRTI development. However, as discussed in my previous review, the analysis of these low-biomass samples leads to some ambiguous results.

 The authors mention that negative controls had extremely low read counts, but do not quantify this or mention what taxa these few reads came from. Even if the counts are low, if they are from the same taxa as our found in other samples, it might be informative. Additionally, as mentioned previously, these negative controls should be included in the SRA upload for the sake of transparency. Finally, the nature of the negative controls, namely that they are PCR controls rather than blank swabs from the same site as the collection, run through the same DNA extraction kit, limits their utility. It is still entirely possible that contamination is present upstream of their negative controls.

 This does not eliminate the value of this study, but should widen the interpretation of some of their findings. Namely, they find an increase in rare taxa in the samples preceding LRTI, and ascribe this to dysbiosis in the nasopharynx. An equally likely interpretation is that LRTI is preceded by reduced bacterial biomass in the nasopharynx, resulting in a higher proportion of sequencing reads coming from contamination. If the authors had information on the biomass of these samples (e.g. qPCR data, or even the DNA concentration post-amplification) it might address whether this phenotype of rare taxa is actually a result of lowered biomass. Otherwise, they should make this interpretation clear.

 Specific revisions:

1. Upload all sequencing data to SRA. Presently only 172 samples are public, which excludes the negative controls and the swabs from the mother.
2. Revise language around the findings from analysis three and four to include the possibility that LRTI samples are lower biomass and have a higher proportion of contamination
3. Explicitly mention the lack of extraction and collection blanks as a limitation. The discussion mentions "our ability to have appropriate control analysis was limited" but this could be clarified for readers not familiar with low-biomass analysis.

Is the work clearly and accurately presented and does it cite the current literature?

Yes

If applicable, is the statistical analysis and its interpretation appropriate?

Yes

Are all the source data underlying the results available to ensure full reproducibility?

Partly

Is the study design appropriate and is the work technically sound?

Partly

Are the conclusions drawn adequately supported by the results?

Yes

Are sufficient details of methods and analysis provided to allow replication by others?

Yes

Reviewer Expertise:

Microbiome, respiratory tract microbiome

I confirm that I have read this submission and believe that I have an appropriate level of expertise to confirm that it is of an acceptable scientific standard, however I have significant reservations, as outlined above.

# Reviewer report: Reviewer response for version 1

**DOI:** 10.21956/gatesopenres.14828.r34801

**Published:** 2023-09-26

**Authors:** Carter Merenstein

**Affiliations:** 1 University of Pennsylvania, Pennsylvania, USA

Major comments:

1. The authors mention sequencing negative controls, but do not include these in any analysis. It would be useful to have some mention of how they were used, and whether there was any overlap between negative controls and experimental samples.
2. Relatedly, I can’t find the negative controls in the SRA upload. These need to be included in the bioproject so that future researchers can also account for potential background.

Minor comments:

1. Figure 3 could use significance bars for clarity. From the surrounding context, the p values in Table 3 must apply to Figure 3, but by eye they don’t seem to match (i.e. in the table Anaerobacillus has a positive LogFC, but the earliest time point in the boxplot seems to be equal or below the healthy control). Significance bars would highlight which groups and which timepoints are actually being compared here.
2. As mentioned in major point 1, Figure 4 would be strengthened by inclusion of the negative control samples to ensure that none of these profiles actually represent the background of the extraction process.
3. The two sections of table 4 (Healthy Infants and LRTI infants) should be presented side by side, rather than one on top of the other, to allow for easy comparison across by taxa. This table also could likely be in the extended data, but that may be more up to the editor.

Is the work clearly and accurately presented and does it cite the current literature?

Yes

If applicable, is the statistical analysis and its interpretation appropriate?

Yes

Are all the source data underlying the results available to ensure full reproducibility?

Partly

Is the study design appropriate and is the work technically sound?

Partly

Are the conclusions drawn adequately supported by the results?

Yes

Are sufficient details of methods and analysis provided to allow replication by others?

Yes

Reviewer Expertise:

Microbiome, respiratory tract microbiome

I confirm that I have read this submission and believe that I have an appropriate level of expertise to confirm that it is of an acceptable scientific standard, however I have significant reservations, as outlined above.

# Response

**Published:** 2024-03-02

**Authors:** Rotem Lapidot

**Affiliations:** Boston Medical Center, USA

We greatly appreciate the comments and advice received from the peer reviewer.  We have attempted to address each of the issues raised and provide below a point-by-point summary of our responses.  In each case we provide the comment verbatim, followed by our responses, heralded by ‘***’ and in italics. 

 Major comments:

 The authors mention sequencing negative controls, but do not include these in any analysis. It would be useful to have some mention of how they were used, and whether there was any overlap between negative controls and experimental samples.

 Relatedly, I can’t find the negative controls in the SRA upload. These need to be included in the bioproject so that future researchers can also account for potential background.

***We thank the reviewer for this important comment. Regrettably, due to very low sequencing reads the control samples were not analyzed further and are no longer available to us. This compromises our ability to control for contamination, and we have addressed this both in the limitations section in the manuscript and in our response to reviewers #1.

**

 Minor comments:

 Figure 3 could use significance bars for clarity. From the surrounding context, the p values in Table 3 must apply to Figure 3, but by eye they don’t seem to match (i.e. in the table Anaerobacillus has a positive LogFC, but the earliest time point in the boxplot seems to be equal or below the healthy control). Significance bars would highlight which groups and which timepoints are actually being compared here.

*Figure 3 shows the relative abundance compared to time of infection. For some infants 8 weeks prior to infection would be the first sample taken, and for other infants, the first sample taken would be 2 weeks prior to the LRTI (and their first sample would be represented in the fourth yellow boxplot). The first boxplot of LRTI infants is the average of infants who had an LRTI 8 weeks after their first sampling and therefor does not match the results in table 3 which provides results of first sample of all 10 LRTI infants.*

**

 As mentioned in major point 1, Figure 4 would be strengthened by inclusion of the negative control samples to ensure that none of these profiles actually represent the background of the extraction process.

***We agree, but unfortunately do not have the data to be included in the figure.

**

 The two sections of table 4 (Healthy Infants and LRTI infants) should be presented side by side, rather than one on top of the other, to allow for easy comparison across by taxa. This table also could likely be in the extended data, but that may be more up to the editor.

***We thank the reviewer for the suggestion and agree that these tables should be presented side by side.

# Reviewer report: Reviewer response for version 1

**DOI:** 10.21956/gatesopenres.14828.r34798

**Published:** 2023-09-26

**Authors:** Frank A Scannapieco

**Affiliations:** 1 University at Buffalo, NY, USA

The authors analyzed nasopharyngeal (NP) samples from a longitudinal, prospective cohort study of 1,981 Zambian mother-infant pairs who underwent NP sampling from 1-week through 14-weeks of age at 2-3-week intervals. The idea was to determine if substantial differences were apparent between the NP microbiome of children destined for a lower respiratory tract infection (LRTI) (N=10) when compared to normal infants (N=30), from essentially birth to 14 weeks. The microbiome was compared using 16S rRNA gene sequencing on the samples, as well as from baseline samples of the infants’ mothers.

 The authors interpret the results to suggest that microbial dysbiosis of the NP microbiome in infants precedes LRTIs. The use of the term dysbiosis implies that the microbiome of infants destined to suffer LRTI showed inherent differences after a few weeks following birth, suggesting that the bacteria present early on somehow direct later risk for infection.

 This study is certainly of interest as there are virtually no longitudinal studies of the NP microbiome of infants who suffer LRTI. The existence of the sample bank built for the Southern Africa Mother-Infant Pertussis study (SAMIPS) conducted in Zambia of infants and their mothers were followed over the first three months of life. The authors are applauded for attempting to glean information about risk factors for LRTI using this biobank.

 Assumptions that the differences in the microbiome seen to precede the LRTI may not entirely explain the child’s risk for LRTI. First, with only 10 samples in the LRTI group, it is quite possible that the microbes identified may not be consistently found using a larger sample size. Also, the rather brief period of observation (14 weeks) also limits the generalizability of the findings.

 LRTI infection is a catch-all diagnosis that includes many etiologic agents, including both viral and bacterial agents. The inability to pinpoint the etiologic agents in this study makes it more difficult to assign risk based on the observed results. The definitions of LTRI used in this study are also fairly non-specific and need to be kept in mind when considering results.

 There is no mention, either in the Background or in the Discussion, that the oral cavity, in addition to the nasopharynx, serves as an important source of microbes into the lower airway. There have been quite a few studies in adults, and far fewer in children, that show that the oral cavity is an important source of lung microbes. At the very least, the oral cavity should be discussed as a possible source of microbes in this context.

 The authors assume that the shifts noted in the proportion of various genera over time in some way influences vulnerability to LRTI. How might this happen? For example, Anaerobacillus was noted as elevated in proportions in subjects with LRTI risk. This is a rather recently described genus of spore forming strict anaerobes. How might this group impact other microbial groups?

 The term dysbiosis may not be the most accurate description for the observations made in this study. The shifting of the flora to be dominated by one or a few bacterial groups at one time and by others later may be “normal”; inclusion of a larger sample size may have attenuated the observed effects. Also, most descriptions of the dysbiotic state note that microbial diversity diminishes with dysbiosis. It seems the opposite is true here, since many more genera were associated with LRTI compared to healthy subjects. Also, limiting analysis to the genus level may have reduced the chance of identifying sub-groups of organisms associated with each state of health. A more granular sequence analysis might be worthwhile.

Is the work clearly and accurately presented and does it cite the current literature?

Partly

If applicable, is the statistical analysis and its interpretation appropriate?

I cannot comment. A qualified statistician is required.

Are all the source data underlying the results available to ensure full reproducibility?

Yes

Is the study design appropriate and is the work technically sound?

Partly

Are the conclusions drawn adequately supported by the results?

Partly

Are sufficient details of methods and analysis provided to allow replication by others?

Yes

Reviewer Expertise:

Oral microbiology; Relationships between oral disease and systemic disease.

I confirm that I have read this submission and believe that I have an appropriate level of expertise to state that I do not consider it to be of an acceptable scientific standard, for reasons outlined above.

# Response

**Published:** 2024-03-02

**Authors:** Rotem Lapidot

**Affiliations:** Boston Medical Center, USA

We greatly appreciate the comments and advice received from the peer reviewer.  We have attempted to address each of the issues raised and provide below a point-by-point summary of our responses.  In each case we provide the comment verbatim, followed by our responses, heralded by ‘***’ and in italics. 

 The authors analyzed nasopharyngeal (NP) samples from a longitudinal, prospective cohort study of 1,981 Zambian mother-infant pairs who underwent NP sampling from 1-week through 14-weeks of age at 2-3-week intervals. The idea was to determine if substantial differences were apparent between the NP microbiome of children destined for a lower respiratory tract infection (LRTI) (N=10) when compared to normal infants (N=30), from essentially birth to 14 weeks. The microbiome was compared using 16S rRNA gene sequencing on the samples, as well as from baseline samples of the infants’ mothers.

 The authors interpret the results to suggest that microbial dysbiosis of the NP microbiome in infants precedes LRTIs. The use of the term dysbiosis implies that the microbiome of infants destined to suffer LRTI showed inherent differences after a few weeks following birth, suggesting that the bacteria present early on somehow direct later risk for infection.

*** Our findings of distinct NP microbiome within days of birth in infants who later developed LRTI in the first 3 months of life, if conformed on larger scale studies, can only suggest an association. We have softened our conclusions accordingly.  In our manuscript we very broadly and shortly discuss hypotheses that could explain this association

*Here, detailed are several hypotheses:*

*1.  The microbiome plays a causative role by one or more of these mechanisms* *(i.e., the microbiome directs later risk of infection):*

*i. The microbiome influences the environment in the nasopharynx (pH, metabolites, antibiotics exerted by the residing bacteria etc.). These environmental changes can indirectly influence a newly acquired pathobiont to be more virulent.*

*ii. The microbiome in the nasopharynx influences host responses to the pathobiont indirectly through immunological pathways.*

*iii. Direct interaction between pathobionts and the microbiome in the NP influencing virulent of the pathobiont (such as in the case with S. pneumoniae and RSV).*

*2. The microbiome is a result of underlying immunological host characteristics* *which are the actual reason for the higher risk of LRTI development.*

*We assume that there is probably more than one explanation for the observed association between the NP microbiome and later risk of LRTI, and there could be several roles the NP microbiome plays in the development of LRTI.*

 This study is certainly of interest as there are virtually no longitudinal studies of the NP microbiome of infants who suffer LRTI. The existence of the sample bank built for the Southern Africa Mother-Infant Pertussis study (SAMIPS) conducted in Zambia of infants and their mothers were followed over the first three months of life. The authors are applauded for attempting to glean information about risk factors for LRTI using this biobank.

*** We thank the reviewer for his kind words. We believe that this unique sample library could provide valuable insights into respiratory health in infants, and we have already completed several other analyses on these samples which are currently submitted for publication.

 Assumptions that the differences in the microbiome seen to precede the LRTI may not entirely explain the child’s risk for LRTI. First, with only 10 samples in the LRTI group, it is quite possible that the microbes identified may not be consistently found using a larger sample size. Also, the rather brief period of observation (14 weeks) also limits the generalizability of the findings.

*** We completely agree with the reviewer’s comment. This is a pilot study, that was aimed to see if there was a signal supporting our hypothesis, with the plan to continue exploring this question on a larger scale if such signal was found.

 LRTI infection is a catch-all diagnosis that includes many etiologic agents, including both viral and bacterial agents. The inability to pinpoint the etiologic agents in this study makes it more difficult to assign risk based on the observed results. The definitions of LTRI used in this study are also fairly non-specific and need to be kept in mind when considering results.

*** Once again, we completely agree with the reviewer’s comment. Given the rarity of LRTI (especially with the modified restricted definition we have used to identify the most severely ill infants) we have a relatively small number of infants. We thus did not further divide into the different assumed etiologies. This is of course one of the limitations of this study (as we discuss in our limitations section), but non the less, biases our results to the null. If certain NP microbiome profiles are associated with specific etiologies of LRTI, then by clumping all etiologies together we might have lost some data suggesting this association. We are very eager to continue to explore this question, and we currently are in the process of analyzing these data sets for possible etiologies.

 There is no mention, either in the Background or in the Discussion, that the oral cavity, in addition to the nasopharynx, serves as an important source of microbes into the lower airway. There have been quite a few studies in adults, and far fewer in children, that show that the oral cavity is an important source of lung microbes. At the very least, the oral cavity should be discussed as a possible source of microbes in this context.

*** We agree with the reviewer that the oral cavity and the oral microbiome is of interest and importance. In the pediatric population (unlike adults) the anatomical niche for the common respiratory pathogens such as S. pneumoniae and H. influenzae is the nasopharynx and not the oral cavity. In this analysis we did not aim to explore the pathogens directly causing the LRTI, but rather explore the respiratory anatomical sight where first interactions with the pathobionts occur. In future studies it would be of interest to collect both NP swabs and oral swabs and explore these associations. A similar analysis of the OP microbiome over time could yield different results, but since we did not sample it, we cannot comment further.

 The authors assume that the shifts noted in the proportion of various genera over time in some way influences vulnerability to LRTI. How might this happen? For example, Anaerobacillus was noted as elevated in proportions in subjects with LRTI risk. This is a rather recently described genus of spore forming strict anaerobes. How might this group impact other microbial groups?

*** It is important to emphasize that we do not assume influences of the microbiome. We are strictly describing our observations. We can try to hypothesize how these observations could be associated with the development of LRTI (as detailed above in the first response), but these would only be hypothesis. Our concept regarding microbiome is that there is significance to the context in which a certain microbe is found. As a simplified example, detecting S. pneumoniae together with Moraxella could have a completely different meaning then detecting S. pneumoniae together with H. influenzae, and therefore hypothesizing regarding a specific microbe would be of little value. We should also emphasize that given the comments we received from other reviewers, these rare genera (such as Anaerobacillus) could also be a result of contamination that we were not able to control for.

 The term dysbiosis may not be the most accurate description for the observations made in this study. The shifting of the flora to be dominated by one or a few bacterial groups at one time and by others later may be “normal”; inclusion of a larger sample size may have attenuated the observed effects. Also, most descriptions of the dysbiotic state note that microbial diversity diminishes with dysbiosis. It seems the opposite is true here, since many more genera were associated with LRTI compared to healthy subjects. Also, limiting analysis to the genus level may have reduced the chance of identifying sub-groups of organisms associated with each state of health. A more granular sequence analysis might be worthwhile.

*** We thank the reviewer for these comments. As suggested, we have taken out the term dysbiosis form the manuscript in appropriate places.

*We and others have noted that the NP microbiome in health is correlated with lower diversity, as opposed to the fecal microbiome where health states are associated with higher diversity. Our finding of what we termed ‘dysbiosis’ referred to the distinct microbiome characteristics we observed in the first sample taken from infants (those who developed LRTI compared to healthy controls) and was not related to the shifting of dominant bacteria over time. When comparing the changes of the NP microbiome during the first 3 months of life between LRTI infants and controls we observed similar succession patterns.*

*We appreciate the comment that a more granular analysis might be useful. Although we have established that species-level classification is made more accurate by metagenomic methods such as PathoScope (Odom et al, 2023), genus level classification is much more reliable. In addition, our results would not be either changed or strengthened by the delineation of specific species (e.g. Dolosigranulum pigrum). So we decided to focus only on the genus level. We have included this justification in the manuscript.*

 ​​​​​​

# Reviewer report: Reviewer response for version 1

**DOI:** 10.21956/gatesopenres.14828.r31973

**Published:** 2022-05-16

**Authors:** Wouter A.A. De Steenhuijsen Piters, Mari-Lee Odendaal

**Affiliations:** 1 Department of Paediatric Immunology and Infectious Diseases, Wilhelmina Children's Hospital, University Medical Center Utrecht, Utrecht, The Netherlands; 2 Centre for Infectious Disease Control, National Institute for Public Health and the Environment, Bilthoven, The Netherlands; 3 Institute for Risk Assessment Sciences (IRAS), Utrecht University, Utrecht, The Netherlands

**Summary:**

 In the original article entitled “ *Nasopharyngeal Dysbiosis Precedes the Development of Lower Respiratory Tract Infections in Young Infants, a Longitudinal Infant Cohort Study*”, Lapidot and co-authors investigated the maturation of the nasopharynx microbiome of 40 Zambian infants, part of whom developed (severe) LRTIs ( *n*=10). This paper is of importance, as it contrasts earlier work, which is largely based on European/Australian cohorts. Regardless, similar signals were identified, with an enrichment of *Dolosigranulum* in healthy infants compared to those developing LRTIs. In addition, the authors suggest *Dolosigranulum* abundance already diminishes prior to LRTI and is found in lower abundance in mothers of children with LRTIs, which is very provocative.

 However, especially these latter analyses need to be further substantiated by statistics, among others appropriately controlling for age in order to draw these conclusions. Apart from that, we are concerned some of the genera related to ‘dysbiosis’ (before/during LRTI; e.g. *Novosphingobium*, *Delftia*, *Anaerobacillus* and *Bacillus*) may not represent true biological signals, instead reflecting background contamination. These species are typically not observed in the nasopharynx (neither in health, nor in disease; see among others Man *et al.*, 2019 ^1^) and instead are reported as part of the ‘reagent kitome’ (Salter *et al.*, 2014 ^2^). More information/in depth analyses are required to make a distinction between true signal/contamination, among others inspecting/reporting blank profiles, running decontam (in case of sufficient controls), and by assessing genera by DNA-isolation run/date. Apart from these main points, we encountered a high number of ‘minor’ points, which largely revolve around unclarities/discrepancies in methods/results, unclear structuring of methods/results, definition of LRTI vs LRTI symptoms and notes on superfluous/repetitive statements/analyses. The paper would benefit from extensive restructuring based on these points in our view.

**Major comments:**

 1. As the authors are aware, nasopharyngeal samples are particularly sensitive to contamination due to their low bacterial density, especially in early life (see among others our previous work; Bosch *et al.*, 2017 ^3^ and De Steenhuijsen Piters *et al.*, 2022 ^4^). This underlines the importance of carefully examining the profiles of NP samples and their blanks. In this current study, the authors included negative controls throughout their laboratory analysis, but discharged the samples after the laboratory steps due to a low number of reads. Although samples were processed at random (allowing authors to correct for batch effects), more global contamination (which may be quite stable in a given lab over time) cannot be corrected for in this manner. In addition, the authors did not report information on bacterial density, which would have been helpful to assess the risk of contamination in these samples (and for example link low biomass with profiles enriched for contaminants).

 A sufficient number of reagent controls and density measurements allows for the use of R-packages like decontam (Davis *et al.*, 2018 ^5^), allowing for filtering of contaminating taxa. We admit this may be challenging in the context of this study, given that low biomass is likely associated with young age and with higher numbers of contaminants. Inspection of decontam-results would therefore be warranted in addition to running decontam in the first place.

 Together, the points raised above open up the discussion on whether contamination has impacted the microbiota profiles reported by the authors. Generally, I believe the average profiles, as well as individual profiles look roughly adequate and in line with previously published literature. However, especially the enrichment of genera including *Novosphingobium*, *Delftia*, *Anaerobacillus* and *Bacillus *in pre-LRTI/LRTI is troublesome, as these are all well-known contaminants (Salter *et al.*, 2014 ^2^) and were not reported in previously published studies (e.g. Teo *et al.*, 2015 ^6^, Kelly *et al.*, 2017 ^7^). Furthermore, among others Extended Figure 1 (healthy infants) shows an early-life (likely low biomass) and highly diverse cluster with *Anaerobacillus *and *Pseudomonas*, which could also reflect possible signs of contamination, rather than true biological signals. This is less problematic, as these genera are not prominently reported as differentially abundant.

 Taken together, we would urge the authors to further assess this issue, for example by further inspecting blank profiles, running decontam (if the number of blanks allows this), assessing DNA density, and assessing samples by DNA-isolation run/DNA-isolation date. If the possibility of contamination cannot be excluded, this should be at least clearly discussed in the paper and conclusions based on possible contaminants should be down-toned.

 2. Some of the conclusions the authors draw are insufficiently supported by their analysis. Among others, although highly provocative, the notion that *Dolosigranulum* abundance already diminishes prior to LRTI is not substantiated by statistical analyses. See for details the minor comments below.

 3. Important clinical information is currently not provided. This includes information on LRTI phenotype, etiology (causative pathogen? Viral data?), clinical information on LRTI symptoms/severity, treatment (antibiotics/immunosuppressants) and co-morbidities.

**Minor comments:**

1. The term ‘dysbiosis’ is generally considered vague. If possible, be specific on what is perturbed compared to healthy controls (e.g. alpha-/beta-diversity, composition of specific taxa, etc).
2. Abstract: ‘Whether these profiles precede the infection or a consequence of it, …’, should be ‘…or are a consequence of it…’.
3. Abstract: it is somewhat misleading to report the total number of participants to this study in the abstract, while microbiota analysis is performed on a (much) smaller subset of these infants. Please report adequate sample size numbers here, if any.
4. Abstract: the methods section could be written a bit more ‘to the point’/shorter.
5. Abstract: ‘… or could be a marker of other pathogenic forces that directly lead to LRTI.’. What is meant by this?
6. Background: second section of the first paragraph on S. pneumoniae seems too detailed. I think the second paragraph also already conveys this message. Consider omitting/shortening this.
7. Background: ‘LRTI is seen … impede or promote LRTI.’; consider simplifying this section a bit. I think the point should be that microbial development is impacted by various host/environmental factors (birth mode/viral infection/nutritional status), which can have direct/indirect impact on pathogen colonization/infection susceptibility.
8. Background: consider shortening the last 2 paragraphs, since especially sample numbers, timing of sampling etc. should be reported in methods.
9. Methods: importantly, was this nested microbiome study part of the initial study plan? What was the primary goal of the SAMIPS study? Could the authors expand on what type of mothers/families were samples; i.e. urbanization level, nutritional status, education level etc.
10. Methods: could the authors be more specific/summarize the (adjusted) WHO-criteria used to determine LRTI?
11. Methods: is the DNA extraction method used based on mechanical lysis/chemical lysis of cells? Especially, chemical lysis could result in an underrepresentation of gram-positives. Is this method benchmarked for use with respiratory samples?
12. Methods: methods for DNA amplification and MiSeq PCR are very detailed, if possible, could the authors refer to other papers using the same protocols? Or state something along the lines of ‘performed in accordance to manufacturer’s instructions’ (if that is the case)?
13. Methods: this paragraph: ‘Sequencing data were processed using QIIME2 (Bolyen et al., 2019) and Pathoscope2 (Hong et al., 2014)’. Samples with less than 10,000 reads were excluded from further analysis.’ should be moved to the ‘Data processing’ section.
14. Methods: this statement ‘To account for reagent … as well as clinical data.’, is a bit odd. Processing in random fashion/blinding is not done to account for contamination in my view. 
15. Methods: information on processing using QIIME is missing. QIIME incorporates many different programs/tools, so please make sure to report the vital tools/parameters used or refer to previous work. Apart from information on filtering and trimming, which was already provided, information on denoising/error correction, merging of paired reads, ASV/OTU-calling, removal of chimeras and taxonomic annotations should be included (or referenced).
16. Methods: why did the authors choose to use PathoScope2 to annotate their 16S-reads? I do not encounter this often; has this been validated in the context of 16S-microbiota analyses? The paper on PathoScope seems to mostly focus on annotation of reads generated through metagenomic sequencing. Why did the authors not use a more standard approach (implemented in QIIME), like a naïve bayseian classifier/DECIPHER to annotate reads?
17. Methods: the authors conducted all the analyses at genus level, it is however unclear why the decision was made to focus on genus and not on individual taxa (ASVs/OTUs). Looking at a lower taxonomic level can be of relevance since specific strains or species within a genus can have a very different function and thus associations with outcomes. We therefore encourage the authors to clearly explain their decision to look at genus level.
18. Methods: the authors used linear regression in their analyses. Among others, linear regression models assume a linear relationship between the predictor (e.g. age; I assume this was modeled as a continuous variable, please specify) and outcome variable (diversity/genus abundance). Did the authors check these assumptions? In figure 1 for instance, age does not appear to be linearly correlated with the relative abundance of the top taxa. We encourage the authors to elaborate on this further, also considering that some genera show a non-linear abundance over time. They could therefore consider fitTimeSeries-analyses (metagenomeSeq-package) or use GAM/spline-based models.
19. Methods: for visualization and modeling, several thresholds for inclusion of genera were used. What was the rationale behind these thresholds? Were these defined post-hoc (after running the analyses) or up front?
20. Methods: p-values of mixed linear models were calculated using ANOVA-tests. Against what model did the authors test their ‘full model’ (including infection status, age, the interaction age:infection status and HIV status)? An empty model, only including an intercept? Also, this model does not account for non-linear microbiota development over time (see previous comment). In addition, given that authors matched for HIV exposure status (according to the results section), why did they add that to their model (while not adding season/maternal age, other factors they matched for)? Could the authors clarify and align information on matching in Methods and Results?
21. Methods: could the authors provide any information on antibiotic usage in these infants (especially around birth/LRTI). Were all infants breastfed?
22. Methods: generally, the description of models is detailed, but also seems repetitive. Please check whether condensing this information a bit more is possible. Also, it is not clear why different modeling frame works were used (e.g. DESEQ2 vs mixed linear models). Also, in DESEQ2 it seems the authors did not account for repeated measures (i.e. subject as random effect).
23. Methods: ‘For each cluster, we performed Fisher’s exact tests to determine whether that cluster was enriched for LRTI samples generally, pre-symptomatic samples, active symptom samples, or HIV-exposed samples.’. It is unclear how these sample-types are defined (particularly ‘active symptom samples’). Are these the same as LRTI-samples? Please clarify. In addition, the authors should consider adjusting for age when assessing enrichment of LRTI-samples in specific clusters, given that these samples are typically collected at older age. This would imply running (mixed) logistic regression models including age.
24. Methods: was the Spearman correlation-analysis between mothers/infants performed on the vector of genus abundances for each mother-infant pair (separately per time point)?
25. Results: first section on definition of LRTIs should be moved to the Methods section.
26. Results/table 1: could any more information on LRTI phenotype be included, what symptoms did infants have, how severe were these LRTIs (severity score), what treatment did infants receive, could the authors share any information on LRTI etiology?
27. Results: ‘A third of infants with LRTI were born to mothers with HIV (receiving anti-retroviral treatment), compared to 40% of infants in the healthy group.’ I do not follow; few lines back the authors describe matching for HIV exposure. Similarly, it seems the authors matched for season, yet there are 2 LRTI infants enrolled in rainy season vs 2 healthy controls enrolled in the same season. According to the 3:1-scheme this should be 6 healthy controls. Was the matching imperfect?
28. Results/table 2; the distinction between symptomatic (define) and non-symptomatic routine visits should be made more clearly in the methods-section.
29. Results/table 2: ‘Diagnosis of LRTI (cough/runny or blocked nose with or without fever AN 0 fast breathing with indrawing of the chest)’. Is ‘AN 0 fast breathing’ a typo?
30. Results/analysis one: I suggest using the term ‘development’ or ‘dynamics’ instead of ‘evolution’.
31. Results/analysis one: ‘… different genera across each age averaged stratum’. Please rephrase.
32. Results/analysis one: please use ‘ *Corynebacterium*’ instead of ‘ *Corynebacteria*’.
33. Results/general: the results on age dynamics in (healthy) NP microbiota composition/diversity lack a measure of effect size. If possible, add this, at least for important findings. Also, as noted in the methods, please check if the assumption that relative abundance is linearly related to age is valid. For example, inspecting scatter plots of relative abundance across age would be helpful.
34. Results/analysis one: ‘However, alpha diversity only reflects the number of dominant genera, and not whether the dominant genera are themselves diverse.’. I politely disagree, dominant genera should be indicated by low richness and low evenness. Consider rephrasing this sentence. I think assessing beta-diversity is valid regardless of alpha-diversity results.
35. Extended Figure 1A: consider removing white lines around bars and annotating the clusters (e.g. MIX-cluster or *Haemophilus* (HAE)-cluster). Addendum: this was done for the full dendrogram-analysis I see; as suggested consider only presenting that analysis instead (i.e. running clusterin once).
36. Extended Figure 1B: explain RCE on x-/y-axis; consider converting to a % of explained variance.
37. Extended Figure 2: also see methods, please define pre-/post- symptom groups more explicitly.
38. Extended Figure 3: in line with previous comments; be clear on LRTI vs LRTI-symptoms and whether there is a difference. Is there anything known on RTI symptoms in healthy controls?
39. Extended Figure 4: I applaud the authors for adding both 2D and 3D NMDS-plots, yet a visualization of the 3D analysis in 2D (i.e. plotting NMDS1 vs NMDS2, NMDS3 vs NMDS2 and NMDS1 vs NMDS3), would be easier to interpret.
40. Results/analysis three: this section (especially the first sections) is generally difficult to follow, it seems partly mixed with methods and seems to discuss several analyses at the same time. Please try to restructure this section, discussing any analyses one-by-one.
41. Figure 2: methods are lacking information on how clinical variables were projected into ordination space (envFit?).
42. Figure 3: why did the authors choose to report these two genera? Please provide a rationale, for example based on other analysis supporting this choice. Also, it would be helpful to know/visualize the number of samples at each time point. Did the authors consider running any statistics on these results?
43. Results/analysis three: the statement ‘Concentrating on the samples taken prior to infection (and prior to antibiotic administration), this analysis confirmed lower relative abundance of *Dolosigranulum*, and higher relative abundance of *Anaerobacillus* in the LRTI infants before their infection.’, although very interesting, is not supported sufficiently by the authors' analyses I believe. Time points prior to infection are likely related to earlier age and therefore higher *Dolosigranulum*, while time points after infections are related to older age and lower *Dolosigranulum*. Therefore, the pattern observed may merely reflect age dynamics. To support the statement the authors are trying to make, it may be interesting to look at time points before/after infection vs and age-matched control samples. Or include age when modeling these effects. Apart from that, from eyeballing this figure, I would also conclude the *Dolosigranulum* dramatically drops upon infection. In fact, it could be both, i.e. lower *Dolosigranulum* prior to infection and a more significant drop after infection. In addition, also given the question of the authors in the discussion (‘But are these microbial profiles a result of the infection? Or were they present before the infection?’), it would make sense to expand/deepen this analysis further, including pathobionts like *Haemophilus*/ *Streptococcus*/ *Moraxella*.
44. Results/analysis four: again, this section includes too many details on methods in my view.
45. Results/analysis four: the authors refer to table 4, yet this seems to not include data on clustering. Should this be Figure 4?
46. Results/analysis four: the authors present many dendrograms/clusterings in their paper (LRTI only, healthy only and all, both clustered based on two indices). If possible, I would advise to combine these analyses and properly name clusters according to the most dominant taxon, so that throughout the paper, the same clusters are discussed. In addition, if clustering based on Frey’s index does not add anything to the message the authors want to convey, I would omit it. Similarly, consider presenting one figure showing per-individual microbiota profiles over time (grouped by LRTI/non-LRTI; currently Extended Figure 3 and 5).
47. Table 5: I suggest to rerun this analysis using cluster/no clusters as response variable and LRTI status + age as predictors.
48. Results/analysis five: very interesting analysis, yet results are presented without any information on effect size. Also, it is not clear what number of genera are tested and whether other genera were also significantly different. Please expand on this analysis a bit further if possible.
49. Results/general: did the authors consider running further analyses on symptomatic/non-LRTI samples?
50. Discussion: make sure all relevant papers are cited, among others Man *et al.* (2019 ^1^) is currently missing, while this to date is one of the largest studies on (severe) LRTI and NP microbiota.
51. Discussion: I think some statements are insufficiently supported by the data; for example ‘… clear evidence of dysbiosis preceding the onset of LRTI.’. This seems based on figure 3, where no statistical support was provided. Also, as said ‘dysbiosis’ is a bit vague, notably, it seems the authors observed specific differentially abundant genera between LRTI vs no LRTI. Same goes for ‘… we observed different microbiome patterns’, this seems based on analysis five, which requires more detail.
52. Discussion: ‘The NP microbiome at time of infection is associated with the risk of development of LRTI and its severity.’. This does not make sense to me, do the authors refer to NP microbiome prior to infection?
53. Discussion: ‘Thus, a similar association between the NP microbiome and risk of respiratory infections is a plausible theory for which there is precedent.’ In fact, this has been shown for NP microbiota in the context of mild infections by our group (Bosch *et al.*, 2017 ^3^ and De Steenhuijsen Piters *et al.*, 2022 ^4^, Nat Microbiol, 2022) and others (Teo *et al.*, 2015 ^6^ and Teo *et al.*, 2018 ^8^).
54. Discussion: discussion on the comparison/possible differences between Zambian children and existing European/Australian cohorts is lacking. This is one of the big strengths of this study; we need data from a wider range of cohorts and this study may contribute to that goal in my view. Another big strength of this study is that data on healthy microbiota are available from children who will develop a severe LRTI (this contrasts among others work from our group, where we assessed longitudinal microbiota data in context of mild respiratory infections).

Is the work clearly and accurately presented and does it cite the current literature?

Partly

If applicable, is the statistical analysis and its interpretation appropriate?

No

Are all the source data underlying the results available to ensure full reproducibility?

Yes

Is the study design appropriate and is the work technically sound?

Partly

Are the conclusions drawn adequately supported by the results?

No

Are sufficient details of methods and analysis provided to allow replication by others?

No

Reviewer Expertise:

Our group's expertise is on early-life development of respiratory microbiota in the context of health and disease. Both reviewers specialise in microbiota data analysis.

We confirm that we have read this submission and believe that we have an appropriate level of expertise to state that we do not consider it to be of an acceptable scientific standard, for reasons outlined above.

## References

[1] : Bacterial and viral respiratory tract microbiota and host characteristics in children with lower respiratory tract infections: a matched case-control study. *The Lancet Respiratory Medicine* .2019;7(5) : 10.1016/S2213-2600(18)30449-1 417-426 10.1016/S2213-2600(18)30449-1 30885620 PMC7172745

[2] : Reagent and laboratory contamination can critically impact sequence-based microbiome analyses. *BMC Biol* .2014;12: 10.1186/s12915-014-0087-z 87 10.1186/s12915-014-0087-z 25387460 PMC4228153

[3] : Maturation of the Infant Respiratory Microbiota, Environmental Drivers, and Health Consequences. A Prospective Cohort Study. *American Journal of Respiratory and Critical Care Medicine* .2017;196(12) : 10.1164/rccm.201703-0554OC 1582-1590 10.1164/rccm.201703-0554OC 28665684

[4] : Early-life viral infections are associated with disadvantageous immune and microbiota profiles and recurrent respiratory infections. *Nat Microbiol* .7(2) : 10.1038/s41564-021-01043-2 224-237 10.1038/s41564-021-01043-2 35058634

[5] : Simple statistical identification and removal of contaminant sequences in marker-gene and metagenomics data. *Microbiome* .2018;6(1) : 10.1186/s40168-018-0605-2 10.1186/s40168-018-0605-2 30558668 PMC6298009

[6] : The infant nasopharyngeal microbiome impacts severity of lower respiratory infection and risk of asthma development. *Cell Host Microbe* .2015;17(5) : 10.1016/j.chom.2015.03.008 704-15 10.1016/j.chom.2015.03.008 25865368 PMC4433433

[7] : The Nasopharyngeal Microbiota of Children With Respiratory Infections in Botswana. *Pediatr Infect Dis J* .2017;36(9) : 10.1097/INF.0000000000001607 e211-e218 10.1097/INF.0000000000001607 28399056 PMC5555803

[8] : Airway Microbiota Dynamics Uncover a Critical Window for Interplay of Pathogenic Bacteria and Allergy in Childhood Respiratory Disease. *Cell Host & Microbe* .2018;24(3) : 10.1016/j.chom.2018.08.005 341-352.e5 10.1016/j.chom.2018.08.005 30212648 PMC6291254

# Response

**Published:** 2024-03-02

**Authors:** Rotem Lapidot

**Affiliations:** Boston Medical Center, USA

We greatly appreciate the comments and advice received from the peer reviewers.  We have attempted to address each of the issues raised and provide below a point-by-point summary of our responses.  In each case we provide the comment verbatim, followed by our responses, heralded by ‘***’ and in italics. 

 In the original article entitled “ *Nasopharyngeal Dysbiosis Precedes the Development of Lower Respiratory Tract Infections in Young Infants, a Longitudinal Infant Cohort Study*”, Lapidot and co-authors investigated the maturation of the nasopharynx microbiome of 40 Zambian infants, part of whom developed (severe) LRTIs ( *n*=10). This paper is of importance, as it contrasts earlier work, which is largely based on European/Australian cohorts. Regardless, similar signals were identified, with an enrichment of *Dolosigranulum* in healthy infants compared to those developing LRTIs. In addition, the authors suggest *Dolosigranulum* abundance already diminishes prior to LRTI and is found in lower abundance in mothers of children with LRTIs, which is very provocative.

*** We thank the reviewer for this clear assessment of the importance of our work

 However, especially these latter analyses need to be further substantiated by statistics, among others appropriately controlling for age in order to draw these conclusions. Apart from that, we are concerned some of the genera related to ‘dysbiosis’ (before/during LRTI; e.g. *Novosphingobium*, *Delftia*, *Anaerobacillus* and *Bacillus*) may not represent true biological signals, instead reflecting background contamination. These species are typically not observed in the nasopharynx (neither in health, nor in disease; see among others Man *et al.*, 2019 ^1^ ) and instead are reported as part of the ‘reagent kitome’ (Salter *et al.*, 2014 ^2^ ). More information/in depth analyses are required to make a distinction between true signal/contamination, among others inspecting/reporting blank profiles, running decontam (in case of sufficient controls), and by assessing genera by DNA-isolation run/date. Apart from these main points, we encountered a high number of ‘minor’ points, which largely revolve around unclarities/discrepancies in methods/results, unclear structuring of methods/results, definition of LRTI vs LRTI symptoms and notes on superfluous/repetitive statements/analyses. The paper would benefit from extensive restructuring based on these points in our view.

*** We thank the reviewer for these assessments and comments. We have updated our manuscript to address the individual points (as detailed below), including issues of statistical significance, contamination, and clarification of methods/results.

**Major comments:**

 1. As the authors are aware, nasopharyngeal samples are particularly sensitive to contamination due to their low bacterial density, especially in early life (see among others our previous work; Bosch *et al.*, 2017 ^3^ and De Steenhuijsen Piters *et al.*, 2022 ^4^ ). This underlines the importance of carefully examining the profiles of NP samples and their blanks. In this current study, the authors included negative controls throughout their laboratory analysis, but discharged the samples after the laboratory steps due to a low number of reads. Although samples were processed at random (allowing authors to correct for batch effects), more global contamination (which may be quite stable in a given lab over time) cannot be corrected for in this manner. In addition, the authors did not report information on bacterial density, which would have been helpful to assess the risk of contamination in these samples (and for example link low biomass with profiles enriched for contaminants).

 A sufficient number of reagent controls and density measurements allows for the use of R-packages like decontam (Davis *et al.*, 2018 ^5^ ), allowing for filtering of contaminating taxa. We admit this may be challenging in the context of this study, given that low biomass is likely associated with young age and with higher numbers of contaminants. Inspection of decontam-results would therefore be warranted in addition to running decontam in the first place.

 Together, the points raised above open up the discussion on whether contamination has impacted the microbiota profiles reported by the authors. Generally, I believe the average profiles, as well as individual profiles look roughly adequate and in line with previously published literature. However, especially the enrichment of genera including *Novosphingobium*, *Delftia*, *Anaerobacillus* and *Bacillus *in pre-LRTI/LRTI is troublesome, as these are all well-known contaminants (Salter *et al.*, 2014 ^2^ ) and were not reported in previously published studies (e.g. Teo *et al.*, 2015 ^6^ , Kelly *et al.*, 2017 ^7^ ). Furthermore, among others Extended Figure 1 (healthy infants) shows an early-life (likely low biomass) and highly diverse cluster with *Anaerobacillus *and *Pseudomonas*, which could also reflect possible signs of contamination, rather than true biological signals. This is less problematic, as these genera are not prominently reported as differentially abundant.

 Taken together, we would urge the authors to further assess this issue, for example by further inspecting blank profiles, running decontam (if the number of blanks allows this), assessing DNA density, and assessing samples by DNA-isolation run/DNA-isolation date. If the possibility of contamination cannot be excluded, this should be at least clearly discussed in the paper and conclusions based on possible contaminants should be down-toned.

*** We thank the reviewers for this important comment. The reviewer’s point about contamination is well taken, and of course such events are known to occur, and we cannot completely eliminate the possibility that contamination events occurred. However, several features of our data suggest that these results are valid. First, the case and comparator samples were processed (DNA extraction, amplicon sequencing) at the same time, in the same lab, using the same batch of reagents. All samples were multiplexed and sequenced on the same flow cell. Note that we did not see these supposed “contamination” organisms in the controls. Contamination should be a consistent event, and so we would have expected to see these microbes in both groups. Second, the longitudinal sampling structure of these data sets also show that these rare organisms were present repeatedly within subjects over repeated independent samplings, and again more abundantly in the case samples, again in a way that is not consistent with contamination events, and which would be nearly impossible to detect in a cross-sectional analysis. Finally, we note that Salter et al., did not mention Anaeorbacillus and Delftia and as common contaminants, the Teo and Kelly studies were conducted in different countries (Teo in particular was in Australia), and they did not report rare microbes (e.g. Kelly only reported OTUs with 1,000+ reads). In addition, we used our PathoScope metagenomic processing pipeline to identify microbes, which we have recently demonstrated to be more accurate than OTU-based methods for taxonomic classifications, especially for less-abundant microbes (Odom et al., Scientific Reports, 2023). Thus, the fact that we are finding additional rare microbes compared to these other studies is expected. However, to further address the reviewer’s concern, we have added further discussion regarding possible contamination, as well as toned down our conclusions with respect to these microbes.

 2. Some of the conclusions the authors draw are insufficiently supported by their analysis. Among others, although highly provocative, the notion that *Dolosigranulum* abundance already diminishes prior to LRTI is not substantiated by statistical analyses. See for details the minor comments below.

*** We have provided responses to the minor concerns below and in the manuscript. For the specific concern with Dolosgranulum, a linear mixed model for pre-LRTI timepoints that accounts for time and individual provides a statistically significant FDR and p-value (<0.001) that the abundance of Dolosogranulum is lower in the infants that later develop LRTI. We interpret this model/p-value as compelling statistical evidence that supports our conclusions.

 3. Important clinical information is currently not provided. This includes information on LRTI phenotype, etiology (causative pathogen? Viral data?), clinical information on LRTI symptoms/severity, treatment (antibiotics/immunosuppressants) and co-morbidities.

*** Table 2 summarizes the clinical data of all infants with LRTI. For this analysis we did not include causative pathogen, but this data is further analyzed in other studies we have done on this population. The logic behind this was to see if regardless of a specific pathogen we can identify infants at risk for severe respiratory infections, and we believe our data suggest so. By definition, all infants enrolled were healthy, with no known immunocompromised states or comorbidities. HIV exposed infants were enrolled only if their mother was treated for HIV during her pregnancy (added clarifying sentence). As well, antibiotic was not prescribed prior to the event of LRTI (some infants were treated at time of LRTI and that is why we did not include analysis of the samples after the LRTI event).

**Minor comments:**

 1. The term ‘dysbiosis’ is generally considered vague. If possible, be specific on what is perturbed compared to healthy controls (e.g. alpha-/beta-diversity, composition of specific taxa, etc).

***We changed the term “dysbiosis” to a more specific description of the observed changes of the microbiome throughout the manuscript, where appropriate.

 2. Abstract: ‘Whether these profiles precede the infection or a consequence of it, …’, should be ‘…or are a consequence of it…’.

***We have added “are” as the reviewers suggested **.**

******3. Abstract: it is somewhat misleading to report the total number of participants to this study in the abstract, while microbiota analysis is performed on a (much) smaller subset of these infants. Please report adequate sample size numbers here, if any.

***We have taken out the general number of participants in the original cohort and included only the number of infants included in this analysis.

**4. Abstract: the methods section could be written a bit more ‘to the point’/shorter.

 *** *We have shortened the methods section in the abstract as the reviewer suggested*

 5. Abstract: ‘… or could be a marker of other pathogenic forces that directly lead to LRTI.’. What is meant by this?

 *** *Changed to “marker of underlying immunological, environmental or genetic characteristics that predispose to LRTI.”*

 6. Background: second section of the first paragraph on S. pneumoniae seems too detailed. I think the second paragraph also already conveys this message. Consider omitting/shortening this.

 *** *We omitted this section as the reviewers suggest.*

 7. Background: ‘LRTI is seen … impede or promote LRTI.’; consider simplifying this section a bit. I think the point should be that microbial development is impacted by various host/environmental factors (birth mode/viral infection/nutritional status), which can have direct/indirect impact on pathogen colonization/infection susceptibility.****

***We have simplified this section to be more concise.

 8. Background: consider shortening the last 2 paragraphs, since especially sample numbers, timing of sampling etc. should be reported in methods.****

***We have shortened the last two paragraphs as the reviewers recommended

 9. Methods: importantly, was this nested microbiome study part of the initial study plan? What was the primary goal of the SAMIPS study? Could the authors expand on what type of mothers/families were sampled; i.e. urbanization level, nutritional status, education level etc.

*** *We have added the requested changes* *.*

**10. Methods: could the authors be more specific/summarize the (adjusted) WHO-criteria used to determine LRTI?****

***We have added the definition of the WHO for pneumonia and the changes we have made to adjust for a more specific diagnosis.

**11. Methods: is the DNA extraction method used based on mechanical lysis/chemical lysis of cells? Especially, chemical lysis could result in an underrepresentation of gram-positives. Is this method benchmarked for use with respiratory samples?

*** *As noted in the methods, we used the EasyMAG system for DNA extraction, which is benchmarked for respiratory samples, and this uses a chemical lysis buffer.*

**12. Methods: methods for DNA amplification and MiSeq PCR are very detailed, if possible, could the authors refer to other papers using the same protocols? Or state something along the lines of ‘performed in accordance to manufacturer’s instructions’ (if that is the case)?

*** *Given that there is no word limit on Gates Open Access, we see no reason to reduce the details about how the PCR and sequencing was performed.*

**13. Methods: this paragraph: ‘Sequencing data were processed using QIIME2 (Bolyen et al., 2019) and Pathoscope2 (Hong et al., 2014)’. Samples with less than 10,000 reads were excluded from further analysis.’ should be moved to the ‘Data processing’ section.****

 *** *We have moved these sentences to the “Data processing” section.*

 14. Methods: this statement ‘To account for reagent … as well as clinical data.’, is a bit odd. Processing in random fashion/blinding is not done to account for contamination in my view. 

*** *We agree that this sentence is confusing and have rewritten it accordingly.*

**15. Methods: information on processing using QIIME is missing. QIIME incorporates many different programs/tools, so please make sure to report the vital tools/parameters used or refer to previous work. Apart from information on filtering and trimming, which was already provided, information on denoising/error correction, merging of paired reads, ASV/OTU-calling, removal of chimeras and taxonomic annotations should be included (or referenced).

*** *While QIIME2 was used for some preliminary analyses, all of our final results were generated with PathoScope.  Thus, we have rewritten this section to eliminate the reference to the older QIIME2 software.*

**

 16. Methods: why did the authors choose to use PathoScope2 to annotate their 16S-reads? I do not encounter this often; has this been validated in the context of 16S-microbiota analyses? The paper on PathoScope seems to mostly focus on annotation of reads generated through metagenomic sequencing. Why did the authors not use a more standard approach (implemented in QIIME), like a naïve bayseian classifier/DECIPHER to annotate reads?

*** *We and others have recently established that metagenomic processing methods such as PathoScope and Kraken provide more accurate taxonomic characterization of microbes in 16S data than OTU/ASV based methods such as QIIME2, DADA2, and Mothur (Odom et. al., Scientific Reports, 2023). The increased accuracy, especially for low-level taxonomies and low-abundance microbes was particularly important for our research goals. Of note, our team developed PathoScope, which is why we selected that metagenomic approach.*

 17. Methods: the authors conducted all the analyses at genus level, it is however unclear why the decision was made to focus on genus and not on individual taxa (ASVs/OTUs). Looking at a lower taxonomic level can be of relevance since specific strains or species within a genus can have a very different function and thus associations with outcomes. We therefore encourage the authors to clearly explain their decision to look at genus level.

*** *A* *lthough we have established that species-level classification is made more accurate by metagenomic methods such as PathoScope (Odom et al, 2023), genus level classification is much more reliable. In addition, our results would not be either changed or strengthened by the delineation of specific species (e.g. Dolosigranulum pigrum). So, we decided to focus only on the genus level. We have included this justification in the manuscript.*

 18. Methods: the authors used linear regression in their analyses. Among others, linear regression models assume a linear relationship between the predictor (e.g. age; I assume this was modeled as a continuous variable, please specify) and outcome variable (diversity/genus abundance). Did the authors check these assumptions? In figure 1 for instance, age does not appear to be linearly correlated with the relative abundance of the top taxa. We encourage the authors to elaborate on this further, also considering that some genera show a non-linear abundance over time. They could therefore consider fitTimeSeries-analyses (metagenomeSeq-package) or use GAM/spline-based models.

*** *We appreciate this concern by the reviewer. We note that our linear mixed models and most other analyses were applied to the log-counts per million (logCPM). The log transformation manages the non-linearity in the abundances quite well and was selected based on appropriate diagnostics as suggested by the reviewer. This approach (linear modeling on logged data) is quite common and standard in these data types, so we feel it does not need special justification in our methods. In addition, because the mixed model on the logged data fits so well, and because our number of longitudinal measurements is relatively small/moderate, we feel that it would be overkill to apply time-series or GAM-based models for these data. For figure 1 in particular, we note that the “stacked” nature of the bar chart accentuates the look of non-linearity in the microbes, and again, what non-linearity is there is easily handled by a log transformation. * 

 19. Methods: for visualization and modeling, several thresholds for inclusion of genera were used. What was the rationale behind these thresholds? Were these defined post-hoc (after running the analyses) or up front?

*** *Yes, thresholds were selected to optimize visual appeal and quality in our results/figures, and were empirically derived/decided. However, in each case we labored over multiple thresholds and were careful that the selection of thresholds did not impact the conclusions to be drawn – but rather were to clarify the results and conclusions.*

 20. Methods: p-values of mixed linear models were calculated using ANOVA-tests. Against what model did the authors test their ‘full model’ (including infection status, age, the interaction age:infection status and HIV status)? An empty model, only including an intercept? *** *We did not use ‘ANOVA-tests’ to calculate p-values for our mixed models. Our Methods section states that we used the “Anova function of the car package” to calculate our p-values; car::Anova is a versatile generic analysis function that can input multiple (>13) distinct R model objects (e,g., lm, glm, multinom, coxph, lme mixed models) and calculates appropriate p-values for the coefficients for these models based on their method/type. We note that car::Anova is a very commonly used function for the calculation of p-values for coefficients for lme mixed model objects in R—and does not use a full/reduced model approach as suggested by the reviewer. We refer the reviewer to the reference in the paper (Weisenberg, et al. 2019) or to the car package user manual (https://cran.r-project.org/web/packages/car/car.pdf) for more details on how car::Anova calculates p-values for mixed models.*

 Also, this model does not account for non-linear microbiota development over time (see previous comment).

***The analyses account for non-linearity through the use of a log transformation (see previous comment)

 In addition, given that authors matched for HIV exposure status (according to the results section), why did they add that to their model (while not adding season/maternal age, other factors they matched for)? Could the authors clarify and align information on matching in Methods and Results?

*** There is a strong body of literature, including our own work (Brennan et al. J Acquir Immune Defic Syndr, 2019, Odom et al., Gates Open Research, 2022), that establishes that HIV-exposure can lead to significant differences in the risk to develop respiratory infections and in the airway microbiome of infants and children. HIV exposure was a significant factor in our data for this paper as well. In contrast, we didn’t observe strong differences in our data based on our other matching factors (season/maternal age, etc). We point out that unless a paired analysis is conducted (e.g. paired t-test), statistically significant matching/confounding variables should always be included as a covariate in a statistical model—hence the inclusion of HIV status in our model. We refer the reviewer to the following reference (or a linear modeling textbook) for more information on why/when matching variables should be included in models: https://www.bmj.com/content/352/bmj.i969 *.*

 21. Methods: could the authors provide any information on antibiotic usage in these infants (especially around birth/LRTI). Were all infants breastfed?

***All infants were deemed healthy when enrolled in the study (and therefore were not given antibiotic by definition). We added a sentence in the study design regarding antibiotic documentation. Since these infants were healthy at enrollment and were followed in the clinic where the study was conducted, we assumed that infants with LRTI did not receive antibiotics prior to their symptoms and that the healthy cohort did not receive antibiotics at all. We also refer to antibiotics’ prescription in our analysis three.

 22. Methods: generally, the description of models is detailed, but also seems repetitive. Please check whether condensing this information a bit more is possible. Also, it is not clear why different modeling frame works were used (e.g. DESEQ2 vs mixed linear models). Also, in DESEQ2 it seems the authors did not account for repeated measures (i.e. subject as random effect).

*** As mentioned above, because Gates does not have a word/page limit, we feel a detailed (albeit repetitive) methods section is preferred to enhance the replicability and reproducibility of our work. We note that DESeq2 was used for two analyses: The analysis of the infant microbiomes at the first timepoint, and 2) the analysis of the maternal microbiomes. DESeq2 was not used for any analyses with repeated measures. Rather, mixed models were used to manage repeated measures in analyses that used longitudinal measures across individuals. Although the DESeq2 and mixed modeling approaches utilize different techniques and error models, they are both valid and standard methods for their respective applications, which is why they were applied in their contexts in the paper.

 23. Methods: ‘For each cluster, we performed Fisher’s exact tests to determine whether that cluster was enriched for LRTI samples generally, pre-symptomatic samples, active symptom samples, or HIV-exposed samples.’. It is unclear how these sample-types are defined (particularly ‘active symptom samples’). Are these the same as LRTI-samples? Please clarify. In addition, the authors should consider adjusting for age when assessing enrichment of LRTI-samples in specific clusters, given that these samples are typically collected at older age. This would imply running (mixed) logistic regression models including age.

*** Each measurement of the microbiome came from a sample that was either HIV exposed or not exposed and came from a child that either experienced an LRTI or not. For the LTRI infants, their points were further classified to their pre-symptomatic (pre-LRTI symptoms), (actively) symptomatic (LRTI symptoms), and post-LRTI. We thank the reviewers for pointing this out and clarified this in the methods. In this unsupervised dimension reduction analysis, we were trying to identify whether a cluster was enriched HIV status, LTRI samples, or pre-, active, post status, or age. We acknowledge that age can be a confounding factor here, and the reviewers make an excellent suggestion on how to adjust for this possible confounding using multivariate analyses. However, here we were just generally exploring the data from a univariate perspective, and did not plan to make any strong statistical conclusions on group membership (or its meaning), therefore for our needs we feel a univariate exploration was sufficient.

**24. Methods: was the Spearman correlation-analysis between mothers/infants performed on the vector of genus abundances for each mother-infant pair (separately per time point)?

*** Yes, the spearman correlation analysis was conducted between the mother and child at the genus level, at only the first time point for the infant. This is already stated in the results section—we have now added this to the methods section.

 25. Results: first section on definition of LRTIs should be moved to the Methods section.

*** We have moved the first section of results to the method section.

**

 26. Results/table 1: could any more information on LRTI phenotype be included, what symptoms did infants have, how severe were these LRTIs (severity score), what treatment did infants receive, could the authors share any information on LRTI etiology?****

*** *The LRTI status was inferred based on whether infants presented with symptoms consistent with WHO’s clinical definition of severe pneumonia, which is based on respiratory rate, fever, and chest wall indrawing, and adapted to the purpose of this study as described. Symptoms of the infants is detailed in table 2, and in general, we identified the 10 infants with the most severe presentation (as discussed in the methods, study design). As we mention, infants were often prescribed antibiotics at the time of diagnosis.  However, our focus is on the events prior to the LRTI.  In our view, the antibiotic exposure impact on the microbiome is a distinct question from whether dysbiosis preceded LRTI, which is the key finding of the paper.*

 27. Results: ‘A third of infants with LRTI were born to mothers with HIV (receiving anti-retroviral treatment), compared to 40% of infants in the healthy group.’ I do not follow; few lines back the authors describe matching for HIV exposure. Similarly, it seems the authors matched for season, yet there are 2 LRTI infants enrolled in rainy season vs 2 healthy controls enrolled in the same season. According to the 3:1-scheme this should be 6 healthy controls. Was the matching imperfect?

 *** We thank the reviewers for pointing out this important point, and we have deleted matching by HIV status. *Matching of some comparisons was performed to the best of our ability, considering that the study was not designed to answer that specific question directly.* 

 28. Results/table 2; the distinction between symptomatic (define) and non-symptomatic routine visits should be made more clearly in the methods-section.****

***The distinction is between scheduled and unscheduled visits, since infants could be symptomatic on a scheduled visit, but could also be seen for an unscheduled visit if they developed symptoms in between study visits. This is detailed in the method section Table 2 summarizes whether an infant had symptoms in each visit, and if they were upper respiratory symptoms or Lower respiratory symptoms, with the definition given in the legend of the figure.

 29. Results/table 2: ‘Diagnosis of LRTI (cough/runny or blocked nose with or without fever AN 0 fast breathing with indrawing of the chest)’. Is ‘AN 0 fast breathing’ a typo?

 *** *We thank the reviewers for pointing out, yes this is a typo (should be AND fast breathing) and was corrected.*

 30. Results/analysis one: I suggest using the term ‘development’ or ‘dynamics’ instead of ‘evolution’.

***The word evolution was replaced with development.

 31. Results/analysis one: ‘… different genera across each age averaged stratum’. Please rephrase.****

***Changed “age averaged stratum” to “age group”.

 32. Results/analysis one: please use ‘ *Corynebacterium*’ instead of ‘ *Corynebacteria*’.****

***We have changed corynebacteria to Corynebacterium.

 33. Results/general: the results on age dynamics in (healthy) NP microbiota composition/diversity lack a measure of effect size. If possible, add this, at least for important findings. Also, as noted in the methods, please check if the assumption that relative abundance is linearly related to age is valid. For example, inspecting scatter plots of relative abundance across age would be helpful.

*** *We added p-values for the microbes that change significantly over time, but did not add an effect size because we feel that effect sizes in this case did not add any value to our narrative. Since we used linear mixed models, with log counts/million to transform the data, the curvilinear relationship of the data has been accounted for statistically.  We apologize for the confusion on this point and have clarified this in the results.*

 34. Results/analysis one: ‘However, alpha diversity only reflects the number of dominant genera, and not whether the dominant genera are themselves diverse.’. I politely disagree, dominant genera should be indicated by low richness and low evenness. Consider rephrasing this sentence. I think assessing beta-diversity is valid regardless of alpha-diversity results.****

***We have deleted this sentence and agree with the reviewer’s comment.

 35. Extended Figure 1A: consider removing white lines around bars and annotating the clusters (e.g. MIX-cluster or *Haemophilus* (HAE)-cluster). Addendum: this was done for the full dendrogram-analysis I see; as suggested consider only presenting that analysis instead (i.e. running clusterin once).

*** We have removed extended figure 1

**36. Extended Figure 1B: explain RCE on x-/y-axis; consider converting to a % of explained variance.

*** *We have removed extended figure 1*

**

 37. Extended Figure 2: also see methods, please define pre-/post- symptom groups more explicitly.

*** We have removed extended figure 2

 38. Extended Figure 3: in line with previous comments; be clear on LRTI vs LRTI-symptoms and whether there is a difference. Is there anything known on RTI symptoms in healthy controls?

*** We have removed extended figure 3

 39. Extended Figure 4: I applaud the authors for adding both 2D and 3D NMDS-plots, yet a visualization of the 3D analysis in 2D (i.e. plotting NMDS1 vs NMDS2, NMDS3 vs NMDS2 and NMDS1 vs NMDS3), would be easier to interpret.

*** We have removed extended figure 4

**40. Results/analysis three: this section (especially the first sections) is generally difficult to follow, it seems partly mixed with methods and seems to discuss several analyses at the same time. Please try to restructure this section, discussing any analyses one-by-one.

***We have shortened and re-orgenized this section

 41. Figure 2: methods are lacking information on how clinical variables were projected into ordination space (envFit?).

*** The Methods section already includes this detail: “… used the vegan’s envfit function to project the age and LRTI status of each sample into the NMDS ordination”

 42. Figure 3: why did the authors choose to report these two genera? Please provide a rationale, for example based on other analysis supporting this choice. Also, it would be helpful to know/visualize the number of samples at each time point. Did the authors consider running any statistics on these results?

*** *We focused on Dolosigranulum because it has previously been identified as a marker of a healthy NP ecosystem, and because our analysis showed a strong signal about changes in the abundance of this pathogen.  For Anaerobacillus, this was a frequent and novel finding within the case infants.*

**

 43. Results/analysis three: the statement ‘Concentrating on the samples taken prior to infection (and prior to antibiotic administration), this analysis confirmed lower relative abundance of *Dolosigranulum*, and higher relative abundance of *Anaerobacillus* in the LRTI infants before their infection.’, although very interesting, is not supported sufficiently by the authors' analyses I believe. Time points prior to infection are likely related to earlier age and therefore higher *Dolosigranulum*, while time points after infections are related to older age and lower *Dolosigranulum*. Therefore, the pattern observed may merely reflect age dynamics. To support the statement the authors are trying to make, it may be interesting to look at time points before/after infection vs and age-matched control samples. Or include age when modeling these effects. Apart from that, from eyeballing this figure, I would also conclude the *Dolosigranulum* dramatically drops upon infection. In fact, it could be both, i.e. lower *Dolosigranulum* prior to infection and a more significant drop after infection. In addition, also given the question of the authors in the discussion (‘But are these microbial profiles a result of the infection? Or were they present before the infection?’), it would make sense to expand/deepen this analysis further, including pathobionts like *Haemophilus*/ *Streptococcus*/ *Moraxella*.

*** *The reviewer is correct that our results are not definitive.  Confirmatory studies will be required to see if this finding can be replicated in other studies/contexts.  We make no claims about the interpretation of Dolosogranulum post LRTI diagnosis as this will certainly be influenced by antibiotic exposure.  That effect is likely to be profound and of course unsurprising, and is not the key finding of our analysis, which focused on events preceding the LRTI event.  We clarify that these models do control for age (see NMDS plots).  The effect of age is controlled for by the inclusion of age-matched comparator samples within the time series analysis.  This seeks to control for potential confounder introduced by the fact that LRTI occurred at different ages, as the reviewer points out.* 

 44. Results/analysis four: again, this section includes too many details on methods in my view.

 *** *We have rearranged this section to include results and moved the methods to the method section*

 45. Results/analysis four: the authors refer to table 4, yet this seems to not include data on clustering. Should this be Figure 4?****

*** *We apologize for not being clearer.  The references are correct as stated, but we have changed the sentence to improve clarity*

**

 46. Results/analysis four: the authors present many dendrograms/clusterings in their paper (LRTI only, healthy only and all, both clustered based on two indices). If possible, I would advise to combine these analyses and properly name clusters according to the most dominant taxon, so that throughout the paper, the same clusters are discussed. In addition, if clustering based on Frey’s index does not add anything to the message the authors want to convey, I would omit it. Similarly, consider presenting one figure showing per-individual microbiota profiles over time (grouped by LRTI/non-LRTI; currently Extended Figure 3 and 5).

*** *We appreciate the reviewer’s comments and have adjusted the results accordingly, and omitted the extended figures for simplification.*

**47. Table 5: I suggest to rerun this analysis using cluster/no clusters as response variable and LRTI status + age as predictors.

 *** *We thank the reviewer for this excellent suggestion. We indeed plan to perform such analysis once we increase our sample size sufficiently to provide meaningful statistical power for such an approach.*

 48. Results/analysis five: very interesting analysis, yet results are presented without any information on effect size. Also, it is not clear what number of genera are tested and whether other genera were also significantly different. Please expand on this analysis a bit further if possible.

*** Regarding maternal data – this is a critical issue and our findings are provoking and intriguing. We chose not to extend on this data in the manuscript and to investigate this topic further separately. We have added a figure of the mothers microbiome (Figure 5).

 49. Results/general: did the authors consider running further analyses on symptomatic/non-LRTI samples? *** *Yes, this was a pilot study, and we are currently working on this sample library looking into different respiratory viruses, HIV exposed infants, mildly symptomatic children, and more.*

 50. Discussion: make sure all relevant papers are cited, among others Man *et al.* (2019 ^1^ ) is currently missing, while this to date is one of the largest studies on (severe) LRTI and NP microbiota.

*** We have added relevant studies, including the suggested important study by Man et al

**

 51. Discussion: I think some statements are insufficiently supported by the data; for example ‘… clear evidence of dysbiosis preceding the onset of LRTI.’. This seems based on figure 3, where no statistical support was provided. Also, as said ‘dysbiosis’ is a bit vague, notably, it seems the authors observed specific differentially abundant genera between LRTI vs no LRTI. Same goes for ‘… we observed different microbiome patterns’, this seems based on analysis five, which requires more detail.

*** We respectfully disagree with this comment. The Figure 3 is only one of many evidentiary features used to make our conclusions: so are the other figures, the mixed model and DESeq2 analyses, etc. There is clear evidence that (in our data) there is a difference between LRTI and control children, and that this precedes the LRTI.

 52. Discussion: ‘The NP microbiome at time of infection is associated with the risk of development of LRTI and its severity.’. This does not make sense to me, do the authors refer to NP microbiome prior to infection?****

***We have rephrased the sentence to correct it. The NP microbiome at time of infection and not prior to it has specific characteristics that are correlated with severity of LRTI.

 53. Discussion: ‘Thus, a similar association between the NP microbiome and risk of respiratory infections is a plausible theory for which there is precedent.’ In fact, this has been shown for NP microbiota in the context of mild infections by our group (Bosch *et al.*, 2017 ^3^ and De Steenhuijsen Piters *et al.*, 2022 ^4^ , Nat Microbiol, 2022) and others (Teo *et al.*, 2015 ^6^ and Teo *et al.*, 2018 ^8^ ).

 *** *We thank the reviewers for pointing this out, we added a sentence and the relevant references*

 54. Discussion: discussion on the comparison/possible differences between Zambian children and existing European/Australian cohorts is lacking. This is one of the big strengths of this study; we need data from a wider range of cohorts and this study may contribute to that goal in my view. Another big strength of this study is that data on healthy microbiota are available from children who will develop a severe LRTI (this contrasts among others work from our group, where we assessed longitudinal microbiota data in context of mild respiratory infections).****

*** We appreciate and thank the reviewers for their comments. We have edited the discussion to reflect these comments.
